# Hierarchical Truncations for Many-Body Expansion Potentials

**DOI:** 10.1021/acs.jctc.6c00471

**Published:** 2026-05-20

**Authors:** Bryce M. Westheimer, Mark S. Gordon, Emilie B. Guidez, Hai Lin

**Affiliations:** † Department of Chemistry, 12226 University of Colorado, Denver, Colorado 80217, United States; ‡ Center for Advanced Computational Molecular Sciences, University of Colorado, Denver, Colorado 80217, United States; § Ames Laboratory, Ames, Iowa 50011, United States; ∥ Department of Chemistry, Iowa State University, Ames, Iowa 50011, United States

## Abstract

In this work, a new strategy to truncate high-order terms in the many-body expansion (MBE) is proposed. This new approach, which we call a hierarchical many-body expansion (HMBE), is based on a hierarchical partition of the system into multitier fragments and can in principle be applied to any large molecular system. Numerical tests on a series of (H_2_O)_64_ structures are presented, demonstrating satisfactory relative energies between the clusters and binding energies of individual clusters compared with full-cluster calculations, with significantly fewer high-order terms computed than conventional MBE. The hierarchical truncation can be augmented by certain many-body terms for fragments at the interface between the partitions (called “Schengen terms”) to further improve accuracy. This work establishes the HMBE scheme as a promising framework to model very large systems (e.g., proteins), which are naturally built on a hierarchical structure.

## Graphical abstract

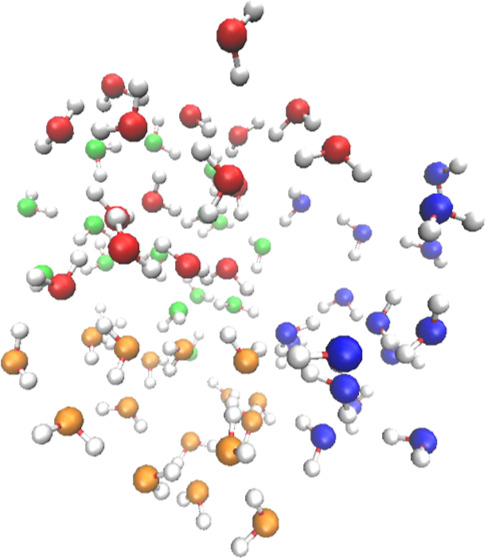

## 1 Introduction

Computational modeling of large systems (a.k.a. containing thousands of atoms) with high-accuracy *ab initio* methods is currently computationally prohibitive due to the steep scaling of these methods, ranging from *N*_bas_^3^ (local density functional theory methods) to *N*_bas_^7^ (coupled-cluster methods), where *N*_bas_ is the number of basis functions. A promising solution is to decompose the system into small pieces (i.e., fragments). Methods utilizing this approach are called fragmentation methods and have been the subject of intense development for the past few decades.^1–9^ Fragmentation methods can leverage highly parallel architectures of modern computers, thereby significantly speeding up the computations.^10,11^ Fragmentation methods are often built on the many-body expansion (MBE), or the many-body analysis (MBA) framework,^12–15^ in which the total energy of a model system is expressed as the sum of the energies of the individual *m* fragments plus the many-body interaction energies among these fragments

1 $$E=\sum_{I=1}^{m}E_{I}+\sum_{I=1}^{m-1}\sum_{J=I+1}^{m}\Delta E_{IJ}+\sum_{I=1}^{m-2}\sum_{J=I+1}^{m-1}\sum_{K=J+1}^{m}\Delta E_{IJK}+...$$

where *E*_*I*_ is the “monomer” energy of fragment *I*

2 $$\Delta E_{IJ}=E_{IJ}-E_{I}-E_{J}$$

is the two-body interaction energy between fragments *I* and *J* that constitute a “dimer” with a dimer energy *E*_*IJ*_

3 $$\Delta E_{IJK}=E_{IJK}-E_{I}-E_{J}-E_{K}-\Delta E_{IJ}-\Delta E_{IK}-\Delta E_{JK}$$

is the three-body interaction energy between fragments *I*, *J*, and *K* that constitute a “trimer” with a trimer energy *E*_*IJK*_, and so on. In general, the fragments do not overlap; but this is not a requirement, and in fact, algorithms have been developed to handle situations in which fragments do overlap.^6,16^

While a full MBE is exact, it suffers from the enormous numbers of high-order terms to be evaluated. The number of *n*-order terms is $\left(\begin{array}{c} m\\ n \end{array}\right)=\frac{m!}{n!\left(m-n\right)!}$ , which corresponds to the number of unique “*n*-mers” that can be formed from *m* fragments. As such, it is often necessary to truncate an MBE. The conventional approach, which is also the most widely adopted, is to neglect *all* interaction energies involving more than *n* fragments, leading to an MBE truncated at the *n* ^th^ order (denoted MBE-*n* in this work). The truncated MBE energy generally converges smoothly to the full MBE energy when a sufficiently large basis set is used.^17^ Other criteria can also be employed. For example, truncation can be done by selectively leaving out certain terms, the estimated energy of which falls below a preset threshold.^18^ This scheme does need a quick and reliable way to compute the approximate energies of the many-body terms, which is not always feasible. A simpler and commonly utilized solution is a distance-based truncation, in which an *n*-body interaction term is omitted if at least one distance between a pair of fragments among the involved *n* fragments is larger than a given preset threshold. Such a treatment is often justified, because the more distant the fragments are from each other, the weaker interactions usually are between them. Of course, exceptions exist, e.g., charged residues with delocalized π orbitals can influence each other even when they are further away from each other than the cutoff distance.

A truncated MBE inevitably introduces “errors” compared with the full MBE, which is exact. It is usually assumed that the high-order terms are small and contribute insignificantly to the total energy. However, although a high-order energy term is generally small in magnitude, the number of high-order terms is very large. While the situation depends on the specific system, one- and two-body energies together can often account for ∼90% of the total energy and ∼95% with three-body energies included.^19,20^ With the inclusion of more high-order terms, a truncated MBE will converge to the full MBE, although the convergence may be slow.

Other formulations of the many-body expansion include the incremental scheme,^15,21–23^ where the localized molecular orbitals of the system are grouped into subunits based on the distances between them, and an MBE (up to third order) is used to compute the correlation energy between these subunits. In a similar spirit, the MBA-based approach by Khire^13^ *et al*. computes the full-cluster energy at the HF level of theory and estimates the MP2 correlation energy in dimers only, showing high computational efficiency and errors between 0.1 and 0.2 mH for the (H_2_O)_16_ and (H_2_O)_17_ structures. Other special treatments, such as the electrostatically embedded many-body (EEMB) methods, have been formulated to accelerate the convergence of MBE.^24,25^ In EEMB, the electronic-structure calculation of each “*n*-mer” is not conducted in the vacuum, as is traditionally done. Instead, the *n*-mer calculation is performed in the presence of a set of background point charges that are assigned to the other atoms in the entire system. Test calculations demonstrated much faster convergence in EEMB toward the full MBE, with insignificant dependence on the choice of point charges, such as Mulliken^26^ or Löwdin^27^ charges. The success of EEMB is not surprising: the *n*-mer calculation is actually performed for the entire system, so that the involved *n* fragments are described by their wave function and the other atoms effectively by point charges.^25^ Therefore, part of the higher-order contributions is implicitly included, speeding up the convergence.

In the present contribution, a new scheme for MBE truncation is proposed, built on the clustering of fragments into tiered substructures. This partitioning scheme is most easily illustrated with proteins, which consists of the following substructures, in hierarchical order: domains → secondary structures (α-helices, β-sheets, and loops) → primary structures (amino-acid residues). Accordingly, one can designate domains as the first-tier fragments, secondary structures as second-tier fragments, and residues as third-tier fragments. The second-tier fragments often interact more strongly with each other if they belong to the same first-tier fragments and less so if not. Exceptions do exist; for example, at the interface between two first-tier fragments (*I*) and (*J*), one 2^nd^-tier fragment from fragment (*I*) may interact more strongly with one 2^nd^-tier fragment from fragment (*J*) than with another remote second-tier fragment from fragment (*I*). Similarly, the third-tier fragments, often (although not always) interact more strongly when they belong to the same second-tier fragments and less so if not. Taking advantage of such hierarchy, the new scheme proposed in this paper systematically neglects certain terms in the MBE according to how the fragments are situated in the tiered structure. The scheme, which we call hierarchical many-body expansion (HMBE), systematically and significantly reduces the number of higher-order terms compared with the conventionally truncated MBE. It is emphasized that, while the natural hierarchical structure of proteins makes the partitioning of the system more intuitive, it is not a requirement to perform HMBE calculations. In this work, the water molecules in a (H_2_O)_64_ cluster^28^ are assembled into two-tiered or three-tiered partitions. The relative energies between different structures of the (H_2_O)_64_ system and the binding energies of individual structures obtained using these four partitions are investigated as proof-of-concept. It is anticipated that applications to proteins will be the subject of a subsequent paper. Furthermore, it is noted that, although this work only focuses on the traditional MBE treatment, where an *n*-mer is computed in the vacuum, it is straightforward to formulate HMBE in the EEMB approach.

## 2 Methods

### 2.1 Hierarchical Partitions of an Entire System

The HMBE method consists in partitioning the system into multiple elementary fragments, which are then assembled at various tiers. For example, Scheme 1 shows the grouping of 64 elementary fragments (in black ovals) into 16 second-tier fragments (in light blue dashed rectangles), which are in turn grouped into four first-tier fragments (in green dashed rectangles). The elementary fragments are the lowest-tier fragments (third-tier in Scheme 1). Note that in the conventional MBE, all fragments are elementary fragments, since the tiered structure is not relevant.

**Scheme 1:**
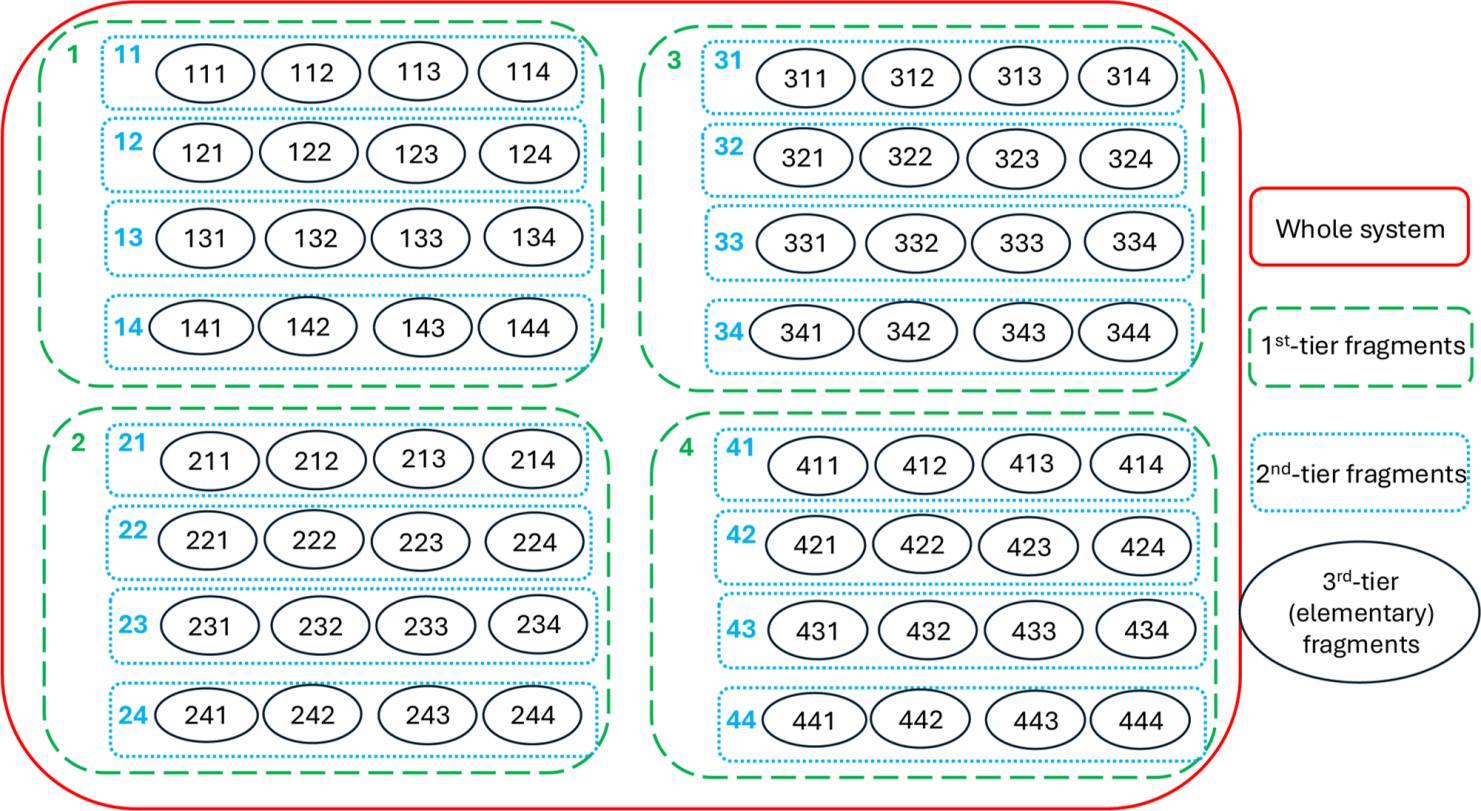
Three-tier HMBE Fragmentation Scheme, where the First Tier Fragments are Represented by Green Dashed Rectangles, Second Tier Fragments by Blue Dotted Rectangles, and third Tier Elementary Fragments by Black Oval Shapes^a^

a The first-, second- and third-tier Fragments are Denoted by Labels in Green, Blue and Black, Respectively.

For simplicity, it is assumed that the fragments within a given tier do not overlap, noting that it is certainly possible to generalize the treatment for overlapping fragments, usually with more complex algorithms. For the same reason, HMBE is presented for fragments without capped atoms (atoms utilized to saturate the dangling bonds in fragments), noting that the method is readily extended to include capped atoms, especially when the *n*-mers are computed in vacuum. Finally, while the HMBE can treat any arbitrary number of tiers, for brevity, we only present the algorithms for a three-tier model in this work. Throughout the remainder of the text, the following notations are employed, as shown in Scheme 1:

- *E*_*I*_ corresponds to the energy of a first-tier fragment (*I*), which is labeled with a capital letter. There are in total *M* first-tier fragments.
- *E*_*Ii*_ corresponds to the energy of a second-tier fragment, which is labeled with two letters: the first letter in capital (*I*) matches the parental (first-tier) fragment and the second letter in lowercase (*i*) identifies the second-tier fragment. The number of second-tier fragments (*i*) in the first-tier fragment (*I*) is *M*_*I*_.
- *E*_Iiμ_ corresponds to the energy of a third-tier fragment, which is labeled with three letters: the first letter in uppercase (*I*) and second letter in lowercase (*i*) correspond to the parental first and second-tier fragments, respectively. The lowercase Greek letter (μ) identifies the third-tier fragment. The number of third-tier fragments (μ) in the second-tier fragment (*Ii*) is *M*_*Ii*_.
- The energy of an *n*-mer is indexed by the monomer index sets, separated by vertical bars for each unique monomer, e.g., *E*_*Ii*|*Jj*_ is the energy of the dimer composed of the second-tier fragments (*Ii*) and (*Jj*), the two-body interaction energy of which is Δ*E*_*Ii*|*Jj*_ = *E*_*Ii*|*Jj*_-*E*_*Ii*_-*E*_*Jj*_.

Although it is convenient to illustrate concepts with equal *M*_*I*_ across all first-tier fragments (*I*) and equal *M*_*Ii*_ across all second-tier fragments (*Ii*), this is obviously not necessary. It is also noted that, while in general the number of fragments increases as one moves down the tiers: *M* ≤ *M*_*I*_ ≤ *M*_*Ii*_, this is not always the case, nor is it a requirement. For example, a helix or loop (second-tier fragment) may be particularly short, with the number of amino acid residues (elementary fragments) smaller than the number of secondary structures presented in the domain.

### 2.2 Hierarchical Truncations: General Consideration

In HMBE, the truncation orders at the first, second, and third tiers are denoted *T*_1_, *T*_2_, and *T*_3_, respectively. A convenient shorthand notation is (*T*_1_,*T*_2_,*T*_3_)-HMBE. We require that *T*_1_ ≤ *T*_2_ ≤ *T*_3_, a requirement in agreement with our intuition that the lower the tier is, the smaller the fragments are, and the more important the high-order interactions would often be. If *T*_1_ = *T*_2_ = *T*_3_ = *n*, HMBE reduces to the conventional MBE truncated at the *n* ^th^ order (i.e., MBE-*n*).

While in general the order of truncation at a given tier is lower than the number of fragments at the tier, i.e., *T*_1_≤*M*, *T*_2_≤max­(*M*_*I*_), and *T*_3_≤max­(*M*_*Ii*_), it is not a requirement. For example, in a particularly short helix or loop, the number of residues may be smaller than the order of truncation at this tier, i.e., *M*_*Ii*_ ≤ *T*_3_ for a particular second tier fragment (*Ii*). In such a case, the effective order of truncation simply equals the number of fragments (*Ii*).

We will illustrate the HMBE algorithm with *T*_1_ = 2,*T*_2_ = 3, and *T*_3_ = 4, i.e., (2,3,4)-HMBE. There are two ways, “top down” and “bottom up,” to construct the HMBE potential energy, which will be described in the next two subsections, respectively.

### 2.3 Hierarchical Truncations: Top Down

In the top-down approach, one moves from the top tier to the bottom tier following the hierarchy of the system. First, let us look at the total energy of the entire system, expressed in terms of the first-tier fragments:

4 $$E=\sum_{I=1}^{M}E_{I}+\sum_{I=1}^{M-1}\sum_{J=I+1}^{M}\Delta E_{IJ}$$

Because *T*_1_ = 2, only the monomer energy *E*_*I*_ and two-body interactions Δ*E*_*IJ*_ remain.

To compute *E*_*I*_ and Δ*E*_*IJ*_ in eq 4, we examine the first-tier fragments, the energies of which involve up to three 2^nd^-tier fragments, because *T*_2_ = 3. The monomer energy of a first-tier fragment (*I*) is given by:

5 $$E_{I}=\sum_{i=1}^{M_{I}}E_{Ii}+\sum_{i=1}^{M_{I}-1}\sum_{i^{\prime }=i+1}^{M_{I}}\Delta E_{Ii|Ii^{\prime }}+\sum_{i=1}^{M_{I}-2}\sum_{i^{\prime }=i+1}^{M_{I}-1}\sum_{i^{\prime \prime }=i^{\prime }+1}^{M_{I}}\Delta E_{Ii\left|Ii^{\prime }\right|Ii^{\prime \prime }}$$

All two-body and three-body terms involving all second-tier fragments within fragment (*I*) are included in *E*_*I*_, the monomer energy of fragment (*I*). The interaction energy Δ*E*_*IJ*_ of a dimer consisting of fragments (*I*) and (*J*) is given by:

6 $$\Delta E_{IJ}=\sum_{i=1}^{M_{I}}\sum_{j=1}^{M_{J}}\Delta E_{Ii|Jj}+\sum_{i=1}^{M_{I}-1}\sum_{i^{\prime }=i+1}^{M_{I}}\sum_{j=1}^{M_{J}}\Delta E_{Ii\left|Ii^{\prime }\right|Jj}+\sum_{i=1}^{M_{I}}\sum_{j=1}^{M_{J}-1}\sum_{j^{\prime }=j+1}^{M_{J}}\Delta E_{Ii\left|Jj\right|Jj^{\prime }}$$

In eq 6, the first term on the right-hand-side (r.h.s.) represents the sum over all two-body interactions between two 2^nd^-tier fragments (*Ii*) and (*Jj*), which constitute first-tier fragments (*I*) and (*J*) respectively. The second term represents the sum over all three-body interactions, with two 2^nd^-tier fragments from fragment (*I*) and the third second-tier fragment from fragment (*J*). The third term is similar to the second term, but with one 2^nd^-tier fragment from fragment (*I*) and the other two 2^nd^-tier fragments from fragment (*J*).

Next, the terms *E*_*Ii*_, $\Delta E_{Ii|Ii^{\prime }}$ , Δ*E*_*Ii*|*Jj*_, $\Delta E_{Ii\left|Ii^{\prime }\right|Ii^{\prime \prime }}$ and $\Delta E_{Ii\left|Ii^{\prime }\right|Jj}$ from eqs 5 and 6 are derived. To this end, we need to move to the second-tier fragments. The energies of the second-tier fragments *E*_*Ii*_ involve up to four elementary third-tier fragments, because *T*_3_ = 4. The monomer energy of a second-tier fragment (*Ii*) is given by:

7 $$E_{Ii}=\sum_{\mu =1}^{M_{Ii}}E_{Ii\mu }+\sum_{\mu =1}^{M_{Ii}-1}\sum_{\mu^{\prime }=\mu +1}^{M_{Ii}}\Delta E_{Ii\mu |Ii\mu^{\prime }}+\sum_{\mu =1}^{M_{Ii}-2}\sum_{\mu^{\prime }=\mu +1}^{M_{Ii}-1}\sum_{\mu^{\prime \prime }=\mu^{\prime }+1}^{M_{Ii}}\Delta E_{Ii\mu \left|Ii\mu^{\prime }\right|Ii\mu^{\prime \prime }}+\sum_{\mu =1}^{M_{Ii}-3}\sum_{\mu^{\prime }=\mu +1}^{M_{Ii}-2}\sum_{\mu^{\prime \prime }=\mu^{\prime }+1}^{M_{Ii}-1}\sum_{\mu^{\prime \prime \prime }=\mu^{\prime \prime }+1}^{M_{Ii}}\Delta E_{Ii\mu \left|Ii\mu^{\prime }\right|Ii\mu^{\prime \prime }|Ii\mu^{\prime \prime \prime }}$$

In eq 7, the energy *E*_*Ii*_ of the second-tier fragment (*Ii*) is written as a function of the one-, two-, three-, and four-body energy terms involving all third-tier fragments constituting the second-tier fragment (*Ii*).

The interaction energies between two 2^nd^-tier fragments (*Ii*) and (*Jj*), Δ*E*_*Ii*|*Jj*_ is given by:

$$\Delta E_{Ii|Jj}=\overset{M_{Ii}}{\underset{\mu =1}{\sum }}\overset{M_{Jj}}{\underset{\nu =1}{\sum }}\Delta E_{Ii\mu |Jj\nu }+\left(\overset{M_{Ii}-1}{\underset{\mu =1}{\sum }}\overset{M_{Ii}}{\underset{\mu^{\prime }=\mu +1}{\sum }}\overset{M_{Jj}}{\underset{\nu =1}{\sum }}\Delta E_{Ii\mu \left|Ii\mu^{\prime }\right|Jj\nu }+\overset{M_{Ii}}{\underset{\mu =1}{\sum }}\overset{M_{Jj}-1}{\underset{\nu =1}{\sum }}\overset{M_{Jj}}{\underset{\nu^{\prime }=\nu +1}{\sum }}\Delta E_{Ii\mu \left|Jj\nu \right|Jj\nu^{\prime }}\right)+\left(\overset{M_{Ii}-2}{\underset{\mu =1}{\sum }}\overset{M_{Ii}-1}{\underset{\mu^{\prime }=\mu +1}{\sum }}\overset{M_{Ii}}{\underset{\mu^{\prime \prime }=\mu^{\prime }+1}{\sum }}\overset{M_{Jj^{\prime }}}{\underset{\nu =1}{\sum }}\Delta E_{Ii\mu \left|Ii\mu^{\prime }|Ii\mu^{\prime \prime }\right|Jj\nu }+\overset{M_{Ii}-1}{\underset{\mu =1}{\sum }}\overset{M_{Ii}}{\underset{\mu^{\prime }=1}{\sum }}\overset{M_{Jj}-1}{\underset{\nu =1}{\sum }}\overset{M_{Jj}}{\underset{\nu^{\prime }=\nu +1}{\sum }}\Delta E_{Ii\mu \left|Ii\mu^{\prime }|Jj\nu \right|Jj\nu^{\prime }}+\overset{M_{Ii}}{\underset{\mu =1}{\sum }}\overset{M_{Jj}-2}{\underset{\nu =1}{\sum }}\overset{M_{Jj}-1}{\underset{\nu^{\prime }=\nu +1}{\sum }}\;\overset{M_{Jj}}{\underset{\nu^{\prime \prime }=\nu^{\prime }+1}{\sum }}\Delta E_{Ii\mu |Jj\nu \left|Jj\nu^{\prime }\right|Jj\nu^{\prime \prime }}\right)$$ 8

On the r.h.s. of eq 8, the first term is the sum over two-body interactions between two 3^rd^-tier fragments, which are from fragments (*Ii*) and (*Jj*), respectively. The second and third r.h.s. terms together represent the sum over three-body interactions with, respectively, two and one 3^rd^-tier fragments from fragment (*Ii*), and the other third-tier fragment(s) from fragment (*Jj*). The last three r.h.s. terms together represent the sum over four-body interactions, with, respectively, three, two, and one 3^rd^-tier fragment from second-tier fragment (*Ii*), and the other third-tier fragment(s) from fragment (*Jj*). Note that *I* = *J* (i.e., the two 2^nd^-tier fragments belong to the same first-tier fragment) is allowed and corresponds to the term $\Delta E_{Ii|Ii^{\prime }}$ in eq 5.

For trimers of second-tier fragments, it is important to recognize that, because *T*_1_ = 2, all three fragments must belong to up to two different first-tier fragments. Let us denote them as (*Ii*), (*Ii* ^′^), and (*Jj*), with the understanding that *I* = *J* corresponds to all three fragments coming from the same first-tier fragment (*I*) ( $\Delta E_{Ii\left|Ii^{\prime }\right|Ii^{\prime \prime }}$ in eq 5). The trimer interaction energy is given by:

9 $$\Delta E_{Ii|Ii^{\prime }|Jj}=\sum_{\mu =1}^{M_{Ii}}\sum_{\nu =1}^{M_{Ii^{\prime }}}\sum_{\lambda =1}^{M_{Jj}}\Delta E_{Ii\mu \left|Ii^{\prime }\nu \right|Jj\lambda }+\left(\sum_{\mu =1}^{M_{Ii}-1}\sum_{\mu^{\prime }=\mu +1}^{M_{Ii}}\sum_{\nu =1}^{M_{Ii^{\prime }}}\sum_{\lambda =1}^{M_{Jj}}\Delta E_{Ii\mu \left|Ii\mu^{\prime }\right|Ii^{\prime }\nu |Jj\lambda }+\sum_{\mu =1}^{M_{Ii}}\sum_{\nu =1}^{M_{Ii^{\prime }}-1}\sum_{\nu^{\prime }=\nu +1}^{M_{Ii^{\prime }}}\sum_{\lambda =1}^{M_{Jj}}\Delta E_{Ii\mu \left|Ii^{\prime }\nu \right|Ii^{\prime }\nu^{\prime }|Jj\lambda }+\sum_{\mu =1}^{M_{Ii}}\sum_{\nu =1}^{M_{Ii^{\prime }}}\sum_{\lambda =1}^{M_{Jj}-1}\sum_{\lambda^{\prime }=\lambda +1}^{M_{Jj}}\Delta E_{Ii\mu \left|Ii^{\prime }\nu \right|Jj\lambda |Jj\lambda^{\prime }}\right)$$

On the r.h.s. of eq 9, the first term is the sum over the three-body interactions between three third-tier fragments, which are from fragments (*Ii*), (*Ii* ^′^), and (*Jj*), respectively. The last three r.h.s. terms together represent the sum over four-body interactions. In each of the four-body interaction terms, one 2^nd^-tier fragment provides two 3^rd^-tier fragments, while the other two 2^nd^-tier fragments each deliver one 3^rd^-tier fragment.

Up to this point, all energy terms have been expressed in terms of the elementary fragments, and the construction of the HMBE potential energy is complete.

### 2.4 Hierarchical Truncations: Bottom Up

In the bottom-up approach, the construction of the HMBE potential energy begins with a conventionally truncated MBE, followed by omitting terms that do not satisfy the HMBE truncations. For (2,3,4)-HMBE, we first write down the conventional MBE-4, which is equivalent to HMBE with *T*_1_ = *T*_2_ = *T*_3_ = 4, or (4,4,4)-HMBE. Next, we delete terms that do not satisfy *T*_1_ = 2 and *T*_2_ = 3.

Let α,β, γ, and δ denote any four elementary fragments (*Ii*μ), (*Jj*ν), (*Kk*λ), and (*Ll*θ). The MBE-4 energy is given by:

10 $$E=\sum_{\alpha }E_{\alpha }+\sum_{\alpha }\sum_{\beta }\Delta E_{\alpha \beta }+\sum_{\alpha }\sum_{\beta }\sum_{\gamma }\Delta E_{\alpha \beta \gamma }+\sum_{\alpha }\sum_{\beta }\sum_{\gamma }\sum_{\delta }\Delta E_{\alpha \beta \gamma \delta }$$

Because *T*_1_ = 2, any Δ*E*_αβγ_ must be omitted if *I*, *J*, and *K* together take more than two different values. For the same reason, any Δ*E*_αβγδ_ must be omitted if *I*, *J*, *K*, and *L* together take more than two different values. Next, because *T*_2_ = 3, any Δ*E*_αβγδ_ must be omitted if *i*, *j*, *k*, and *l* together take more than three different values. For implementations, this can be done easily by setting the value to 0 for the terms to be omitted from the conventional MBE, thus requiring minimal modifications to most existing MBE codes.

### 2.5 Hierarchical Truncations: Scalability

Consider a model system consisting of *m* elementary fragments. Then, for a conventional MBE truncated at the *n*-th order, the number of terms in the expansion to be evaluated is:

11 $$N_{term}^{MBE}\left(m,n\right)=\sum_{t=1}^{n}\left(\begin{array}{l} m\\ t \end{array}\right)$$

where $\left(\begin{array}{c} m\\ t \end{array}\right)=\frac{m!}{t!\left(m-t\right)!}$ denotes “*m* choose *t*”. When *m*≫*n*, the number of terms is dominated by that of the highest *t*-th order, $\left(\begin{array}{c} m\\ n \end{array}\right)$ . In the example of the (2,3,4)-HMBE with 64 elementary fragments (Scheme 1), *m* = 64≫*n* = 4, and the scalability with respect to *m* is:

12 $$O\left(m\right)∼\left(\begin{array}{l} m\\ 4 \end{array}\right)=\frac{m!}{4!\left(m-4\right)!}=\frac{m\left(m-1\right)\left(m-2\right)\left(m-3\right)}{24}∼m^{4}$$

Because many high-order terms are omitted in HMBE, less terms need to be evaluated. For *m*≫*T*_3_ = 4, the number of terms is again dominated by that of the highest *T*_3_-th order, i.e., the fourth order. For simplicity, assume that at any given tier, each fragment contains the same number of fragments in the next lower tier. That is, there are *M* first-tier fragments, each of which contains *M*_*I*_ second-tier fragments, each of which in turn contains *M*_*Ii*_ third-tier fragments. In total, there are *MM*_*I*_ *M*_*Ii*_ = *m* third-tier fragments. The number of fourth order terms *N*_fourth_^(2,3,4)–HMBE^ in (2,3,4)-HMBE is

13 $$N_{4th}^{\left(2,3,4\right)-HMBE}\left(M,M_{I},M_{Ii}\right)=N_{4th}\left(one\;1^{st}-tier,one\;2^{nd}-tier\right)+N_{4th}\left(one\;1^{st}-tier,\;two\;2^{nd}-tiers\right)+N_{4th}\left(one{\;1}^{st}-tier,\;three2^{nd}-tiers\right)+N_{4th}\left(two\;1^{st}-tiers,\;two\;2^{nd}-tiers\right)+N_{4th}\left(two{\;1}^{st}-tiers,\;three\;2^{nd}-tiers\right)$$

where the notations in the parentheses of the terms on the r.h.s. of eq 13 characterize how the third-tier elementary fragments are selected from the first- and second-tier fragments for each term. Specifically, *N*_fourth_(one 1^st^-tier, one 2^nd^-tier), *N*_fourth_(one 1^st^-tier, two 2^nd^-tiers), and *N*_fourth_(one 1^st^-tier, three 2^nd^-tiers) indicate that the four 3^rd^-tier elementary fragments are selected from one, two, and three different 2^nd^-tier fragments of the *same* first-tier fragment, respectively. Similarly, *N*_fourth_(two 1^st^-tiers, two 2^nd^-tiers) and *N*_fourth_(two 1^st^-tiers, three 2^nd^-tiers) indicate that the four 3^rd^-tier elementary fragments are selected from two and three different 2^nd^-tier fragments, respectively. The detailed expressions of these terms are given in the Supporting Information.

When *M*≫1,*M*_*I*_≫1, and *M*_*Ii*_≫1, approximately, the different terms in eq 13 can be expressed as

14 $$N_{term}^{\left(2,3,4\right)-HMBE}\left(M,M_{I},M_{Ii}\right)∼MM_{I}M_{Ii}^{4}+MM_{I}^{2}M_{Ii}^{4}+MM_{I}^{3}M_{Ii}^{4}+M^{2}M_{I}^{2}M_{Ii}^{4}+M^{2}M_{I}^{3}M_{Ii}^{4}$$

In eq 14, the leading term is the last term

15 $$N_{term}^{\left(2,3,4\right)-HMBE}\left(M,M_{I},M_{Ii}\right)∼M^{2}M_{I}^{3}M_{Ii}^{4}$$

The effective scaling of HMBE depends on the choice of *M*, *M*_*I*_, and *M*_*Ii*_, which satisfy the constraint *MM*_*I*_ *M*_*Ii*_ = *m*. For example, if M = 1, *M*_*I*_ = 1, and *M*_*Ii*_ = *m*, it will be *m* ^4^. This is just the same as conventional MBE truncated at the fourth order, because there is only one 1^st^-tier fragment, which consists of only one 2^nd^-tier fragment, and truncations at the first and second tiers have no impact. On the other hand, if M = *m*, *M*_*I*_ = 1, and *M*_*Ii*_ = 1, the scaling will be *m* ^2^. This is just the same as conventional MBE truncated at the second order, because all first-tier fragments are effectively elementary fragments. In general, the effective scaling order *n*_eff_ is expected to vary between *T*_1_ and *T*_3_.

The conventional MBE-4, or (4,4,4)-HMBE scales as *M* ^4^ *M*_*I*_^4^ *M*_*Ii*_^4^. Therefore, the computational cost in (2,3,4)-HMBE is reduced roughly by a factor of *M* ^2^ *M*_*I*_. An immediate and interesting conclusion is that, for a given total number of *m* elementary fragments, the more first-tier fragments (large *M*), the fewer terms in HMBE. A large number of second-tier fragments (large *M*_*I*_) also helps, albeit less effective than *M*. This is not surprising, because the impact of the HMBE truncation is the most severe at the first-tier, and becomes less so as one moves down the hierarchy: *T*_1_≤*T*_2_≤*T*_3_.

### 2.6 Hierarchical Truncations: Augmented Schengen Terms

As mentioned in the Introduction, at the interface where first-tier fragments meet, many-body terms involving second-tier fragments, each from a different first-tier fragment, may be significant. An example will be a three-body term involving second-tier fragments (*Ii*), (*Jj*), and (*Kk*) at the interface between the first-tier fragments (*I*), (*J*), and (*K*). These second-tier many-body terms are named second-tier “Schengen terms” after the village and commune of Schengen, where three countries (France, Germany, and Luxembourg) meet (Figure 1). Similarly, third-tier Schengen terms may be important at the interface between second-tier fragments. Depending on the truncation orders, however, some of these terms may be excluded in an HMBE. For example, if the truncation order *T*_1_ is equal to 2, all second-tier three-body Schengen terms will be missed. Although the number of Schengen terms is usually quite small compared with the total number of many-body terms in an HMBE, their absence may negatively impact the accuracy of the HMBE.

**Figure 1:**
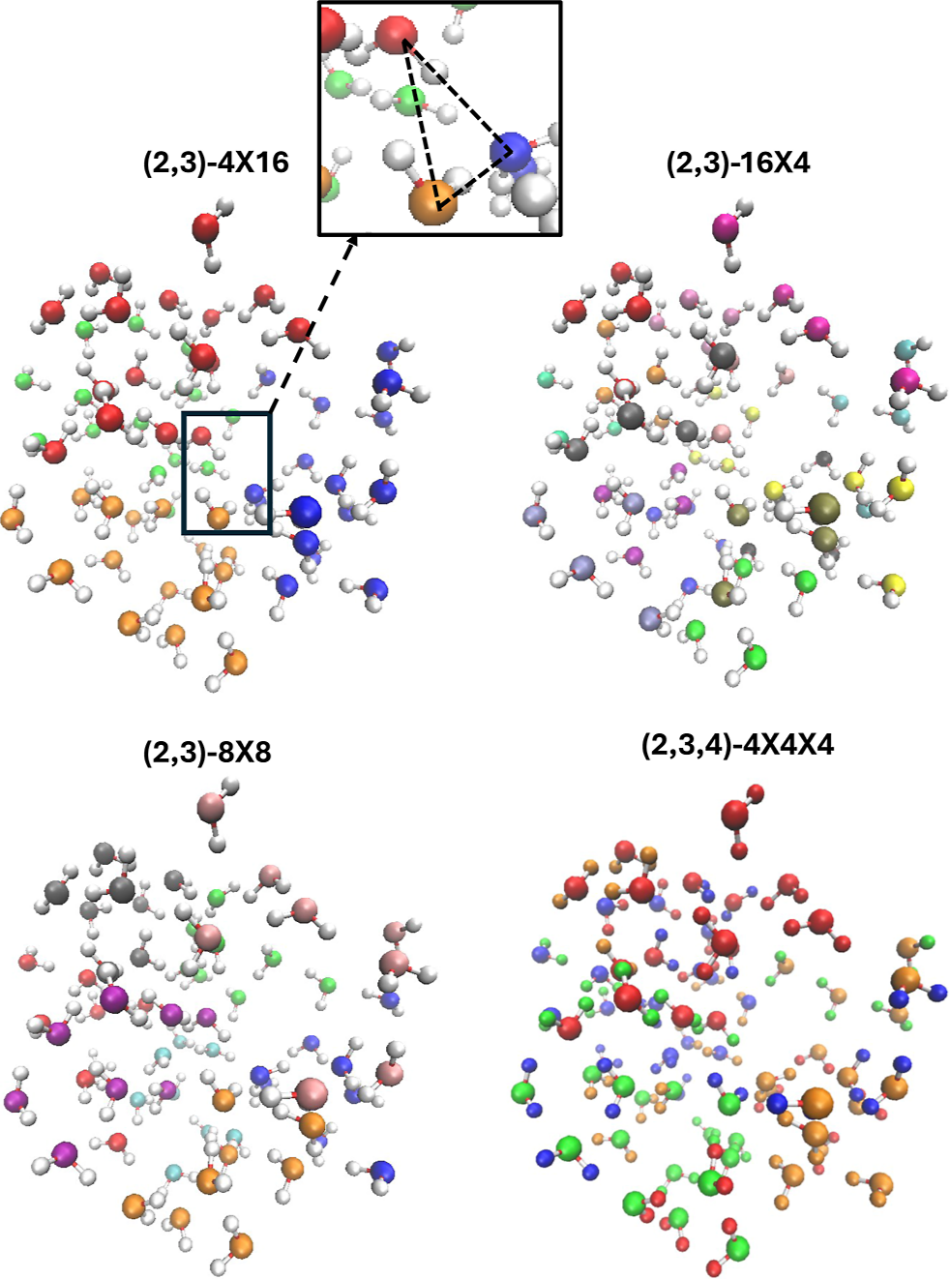
(2,3)-HMBE partitions (4-by-16, 16-by-4, and 8-by-8) and the (2,3,4)-HMBE partition (4-by-4-by-4) of structure 1 of the (H_2_O)_64_ cluster. In each partition, water molecules belonging to the same first-tier fragment are indicated by oxygen atoms of the same color. In the three-tier partition, water molecules belonging to the same second-tier fragment are indicated by hydrogen atoms of the same color. The water trimer indicated by the dotted black line in the inset designates one of the second-tier three-body Schengen terms.

We therefore propose augmenting an HMBE with the inclusion of certain additional higher-order Schengen terms. These terms are selected and added to HMBE based on carefully chosen predefined criteria. In this case, the cutoff scheme in the fragment molecular orbital (FMO) theory, is considered. This scheme was established in the approximation of electrostatic potentials.^29^ It is an elaborate scheme that considers all pairwise distances between two fragments. For water clusters, the oxygen–oxygen (*R*_OO_), the four oxygen–hydrogen (*R*_OH_), and the four hydrogen–hydrogen (*R*_HH_) distances between two water molecules are measured and divided by the sum of van der Waals (vdW) radii for the specific atom pair. The resulting unitless reduced distances (e.g., *R*_OO_^′^, *R*_OH_^′^, and *R*_HH_^′^ for water molecules) are compared with preset cutoff thresholds to decide which many-body electron density matrices (i.e., electron density matrices involving any given specific number of fragments) should be included in the FMO calculations. More details can be found in the literature.^29,30^ Here, this cutoff scheme is adopted for the admission of the Schengen terms, i.e., the Schengen terms are accepted into the HMBE if they satisfy the FMO cutoff thresholds.

## 3 Computations

As proof of concept, the HMBE method is tested on nine (H_2_O)_64_ structures, extracted from liquid water molecular dynamics (MD) simulations in ref 28. It is noted that a total of 10 structures had been provided in the cited reference, but one structure showed some water molecules within unreasonably close proximity of each other, giving rise to an unreasonably large energy compared with the others in full-cluster calculations. This structure was therefore omitted from our analysis. For the other nine structures, the water molecules are partitioned using a constrained k-means clustering algorithm, which has been implemented in a standalone k-means-constrained Python package.^31^ Four different partitions are attempted, which are described below (see also Figure 1 and Table S9 in the Supporting Information). First, three two-tier HMBE partitions with *T*_1_ = 2and *T*_2_ = 3, i.e., (2,3)-HMBE, are considered:

- (2,3)-4 × 16: The molecules are grouped into four first-tier fragments, each of which consists of 16 water molecules as second-tier fragments.
- (2,3)-16 × 4: The molecules are grouped into 16 first-tier fragments, each of which consists of four water molecules as second-tier fragments.
- (2,3)-8 × 8: The molecules are grouped into eight first-tier fragments, each of which consists of eight water molecules as second-tier fragments.

Next, one three-tier HMBE partition with *T*_1_ = 2 and *T*_2_ = 3, and *T*_3_ = 4 i.e., (2,3,4)-HMBE is considered:

- (2,3,4)-4 × 4 × 4: The molecules are grouped into four first-tier fragments, each consisting of four 2^nd^-tier fragments. These second-tier fragments are composed of 4 water molecules as third-tier fragments. This partition is the same as the one illustrated in Scheme 1.

These four HMBE calculations are compared with conventional MBE truncations after the third order (MBE-3) and with full-cluster calculations. Each (2,3)-HMBE was also augmented with second-tier three-body Schengen terms according to the FMO cutoff thresholds for the reduced distances: *R*_OO_^′^ ≤ 2.0, *R*_OH_^′^ ≤ 2.5, and *R*_HH_^′^ ≤ 2.0; the vdW radii are set to 1.52 Å for the oxygen and 1.20 Å for the hydrogen in water, respectively.^30^ The Schengen-augmented (2,3)-4 × 16 HMBE is denoted (2,3)-S-4 × 16, and similarly (2,3)-S-8 × 8 and (2,3)-S-16 × 4 are defined. For the three-tiered HMBE partition, the same second-tier three-body Schengen contributions are considered, without third-tier four-body Schengen terms, and the results are denoted (2,3,4)-S-4 × 4 × 4. The total number of many-body terms computed for these structures are listed in Table S1 of the Supporting Information.

The energies for all systems are computed at the Hartree–Fock^32^ level of theory with the aug-cc-pVDZ^33,34^ basis set. This level of theory is chosen to make full-cluster calculations achievable in our system. For the sake of consistency, all calculations are performed using Psi4,^35^ where QCEngine^36,37^ and QCElemental^38^ are used for interfacing codes to Psi4. The HMBE calculations are performed with a small standalone Python code, while conventional MBE calculations are performed with the QCManyBody^39^ package. In all HMBE, MBE-3 and full-cluster calculations, all standard defaults for Psi4 calculations, such as density-fitting in the SCF calculation, are used in all cases.

For all HMBE, MBE-3 and full-cluster calculations, the relative energy of structure *c* (*c* = 1, 2, ..., 9) with respect to structure 1 (the lowest energy structure) is evaluated as:

16 $$E_{c}^{rel}=E_{c}-E_{1}$$

The mean signed error (MSE) and mean unsigned error (MUE) of the relative energies for a given HMBE partition are calculated by averaging *E*_*c*_^rel^ and |*E*_*c*_^rel^|, respectively, over all 9 structures. The MUE and MSE per elementary fragment (denoted MUE/f and MSE/f) are the total MUE and MSE divided by the number of elementary fragments (64 in this case), respectively.

For the full cluster calculations, the binding energy Δ*E* of a given structure is computed as the difference between the total energy of the system and the sum of the one-body energy terms (a.k.a. the energy of the monomer):

17 $$\Delta E=E_{total}-\sum E_{monomer}$$

For HMBE and MBE-3 calculations, Δ*E* is the sum of the many-body interactions energy terms computed. It is noted that all n-mer energies are computed in vacuo using the geometry of the n-mer in the (H_2_O)_64_ structure. Similar to the analysis of the relative energies, the MSE, MUE, MSE/f, and MSE/f are defined.

## 4 Results and Discussion

### 4.1 HMBE Relative Energies

The absolute energies for all structures are collected in Table S2 and Table S3 of the Supporting Information. Structure 1 is the lowest in energy in all HMBE, MBE-3 and full-cluster calculations. The relative energies with respect to structure 1 are illustrated in Figure 2, with the details in Table S4 in the Supporting Information. Note that these structures are snapshots extracted from MD simulations rather than optimized local minima, and not surprisingly, their energies fluctuate substantially. The MUE/f of the relative energy is shown in Figure 3A as a function of the number of *n*-mers computed and in Figure 3B as a function of the percentage of trimers computed, respectively. The MUE and MSE of the relative energies are reported in Table S5 of the Supporting Information.

**Figure 2:**
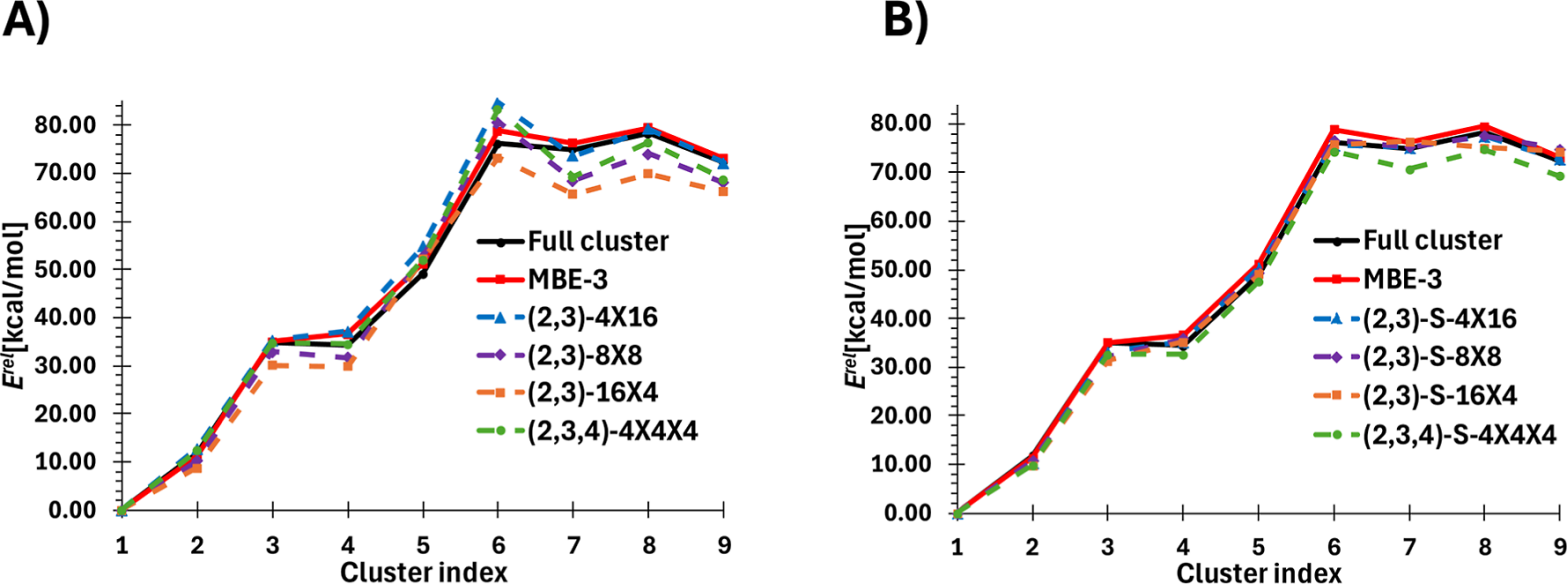
(**A**) Relative energies with respect to Structure 1 for HMBE partitions (2,3)-4 × 16, (2,3)-8 × 8, (2,3)-16 × 4, and (2,3,4)-4 × 4 × 4. (**B**) Schengen-corrected HMBE partitions (2,3)-S-4 × 16, (2,3)-S-8 × 8, (2,3)-S-16 × 4, and (2,3,4)-S-4 × 4 × 4. The reference MBE-3 and full-cluster data are shown for comparison.

**Figure 3:**
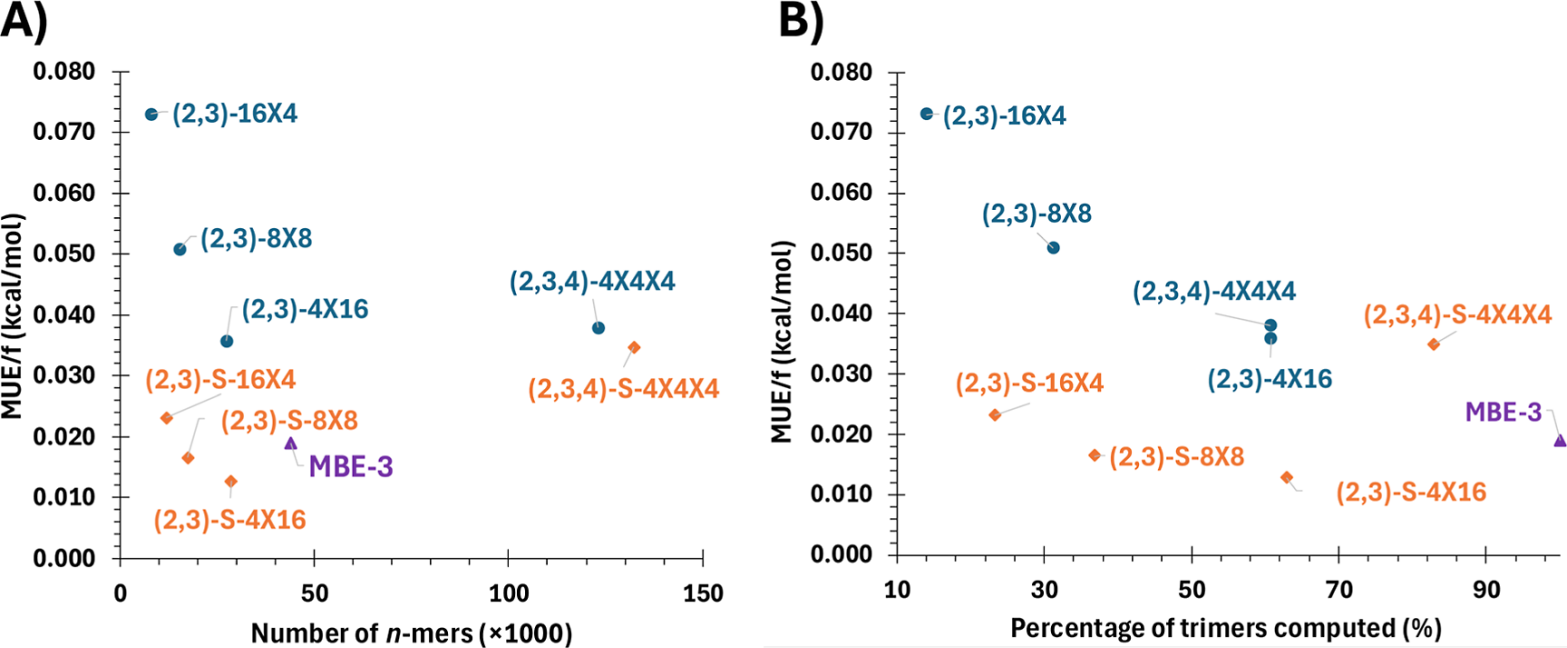
MUE/f of the relative energy with respect to full-cluster calculations against (**A**) the total number of terms computed and (**B**) the percentage of trimers computed. Regular HMBE calculations are shown by blue circles, Schengen-augmented HMBE calculations by orange diamonds, and conventional MBE-3 by a purple triangle.

All HMBE partitions investigated show overall good agreement with the full-cluster calculations, which are in qualitative agreement with the DFT relative energies reported in ref 28. The HMBE error in the relative energy compared to the full cluster calculation tends to be larger for clusters 6–9, which are within 6 kcal/mol of each other in energy. Nevertheless, the HMBE method reproduces qualitatively the potential energy surface of the system. The accuracy of the two-tier HMBE calculations increases in the order: (2,3)-16 × 4 < (2,3)-8 × 8 < (2,3)-4 × 16, with a MUE/f decreasing from 0.073 kcal/mol for the (2,3)-16 × 4 partition to 0.036 kcal/mol for the (2,3)-4 × 16 partition. This behavior appears to correlate with the increasing number of three-body terms included in the MBE, as shown in Figure 3B, consistent with the fact that 3-body terms are essential to obtain accurate energies for water systems.^40,41^ For the three-tier partition (2,3,4)-4 × 4 × 4, the MUE/f is 0.038 kcal/mol, comparable with the (2,3)-4 × 16 partition, as the number of trimers calculated is the same for both (Figure 3B). The addition of Schengen terms further improves the relative energies, with a reduced MUE/f of 0.013, 0.017, and 0.023 kcal/mol for the (2,3)-S-4 × 16, (2,3)-S-8 × 8, and (2,3)-S-16 × 4 partitions, respectively. These values are comparable to the MBE-3 values (MUE/f of 0.019 kcal/mol), even though HMBE-S computes significantly less terms than MBE-3. For instance, for the (2,3)-S-4 × 16 partition, ∼28,000 energy terms are computed versus ∼43,000 terms for the full MBE-3 calculation. It is also noted that the Schengen terms only represent 3.5% of all trimers computed for the (2,3)-S-4 × 16 partition. This number rises to 15.2% and 39.9% for the (2,3)-S-8 × 8 and (2,3)-S-16 × 4 partitions, respectively. The rising contribution of these 3-body Schengen terms correlates with the larger decrease of the MUE/f observed compared to the regular HMBE calculations that do not include these terms. The three-tier (2,3,4)-S-4 × 4 × 4 HMBE, which includes the three-body Schengen terms at the second-tier level (representing about 27% of all trimers), shows only a modest improvement of the MUE per elementary fragment compared to the (2,3,4)-4 × 4 × 4 calculation, with a value of 0.035 kcal/mol. While more trimers are calculated (which we would expect to increase accuracy), only 15% of the four-body terms (tetramers) are accounted for, and some of these terms might be repulsive. For this system, the inclusion of these tetramers likely offsets the benefit of adding more trimers. It is emphasized that increasing the number of HMBE tiers does not necessarily imply an increase in accuracy, especially for small systems like the one studied here. Furthermore, similar to the 2-tier systems, results may vary based on how the system is partitioned. A detailed study of 3-tier partitions will be the subject of another publication.

It is also interesting to note that, while one can view the treatment of Schengen terms as an “augmentation” to improve the accuracy of an HMBE, e.g., to improve (2,3)-S-HMBE toward (3,3)-HMBE (a.k.a. MBE-3), the treatment can also be conversely regarded as a “screening” tool to reduce the computational costs of an MBE, e.g., to trim down the number of terms of MBE-3. This is particularly obvious from the point of view of the bottom-up implementation.

### 4.2 HMBE Binding Energies

The total binding energy between the 64 water molecules in all 9 structures for all HMBE, MBE-3 and full-cluster calculations is shown in Figure 4. The numerical values are included in Table S6 of the Supporting Information. Like the relative energies, HMBE captures the correct trend in binding energies, with a significant improvement when the Schengen terms are included. The data show that the two-tier partitions tend to overestimate the binding energy compared to the full-cluster calculation. This overestimation decreases in the order (2,3)-16 × 4> (2,3)-8 × 8 > (2,3)-4 × 16, and reaches on average 17.08, 8.94, and 5.43 kcal/mol, respectively. With the inclusion of the Schengen terms, this overestimation further decreases to 6.15, 4.85, and 4.43 kcal/mol, respectively. It is noted that all these three (2,3)-S HMBE calculations show excellent agreement with MBE-3, with an MUE of 2.34, 1.04, and 0.62 kcal/mol for (2,3)-S-16 × 4, (2,3)-S-8 × 8, and (2,3)-S-4 × 16, respectively. On the other hand, the three-tier (2,3,4)-4 × 4 × 4 partition underestimates the binding energy of all but two structures (structures 5 and 6) compared with full-cluster calculations. With the Schengen augmented (2,3,4)-S-4 × 4 × 4 partition, all binding energies are underestimated by 2.74 kcal/mol on average compared to full cluster calculations. It is noted that the HMBE method (like the general MBE method) is not variational. Therefore, the HMBE energy can be lower than the full cluster energy, as shown for the (2,3,4)-S-4 × 4 × 4 partition in Table S2 of the Supporting Information.

**Figure 4:**
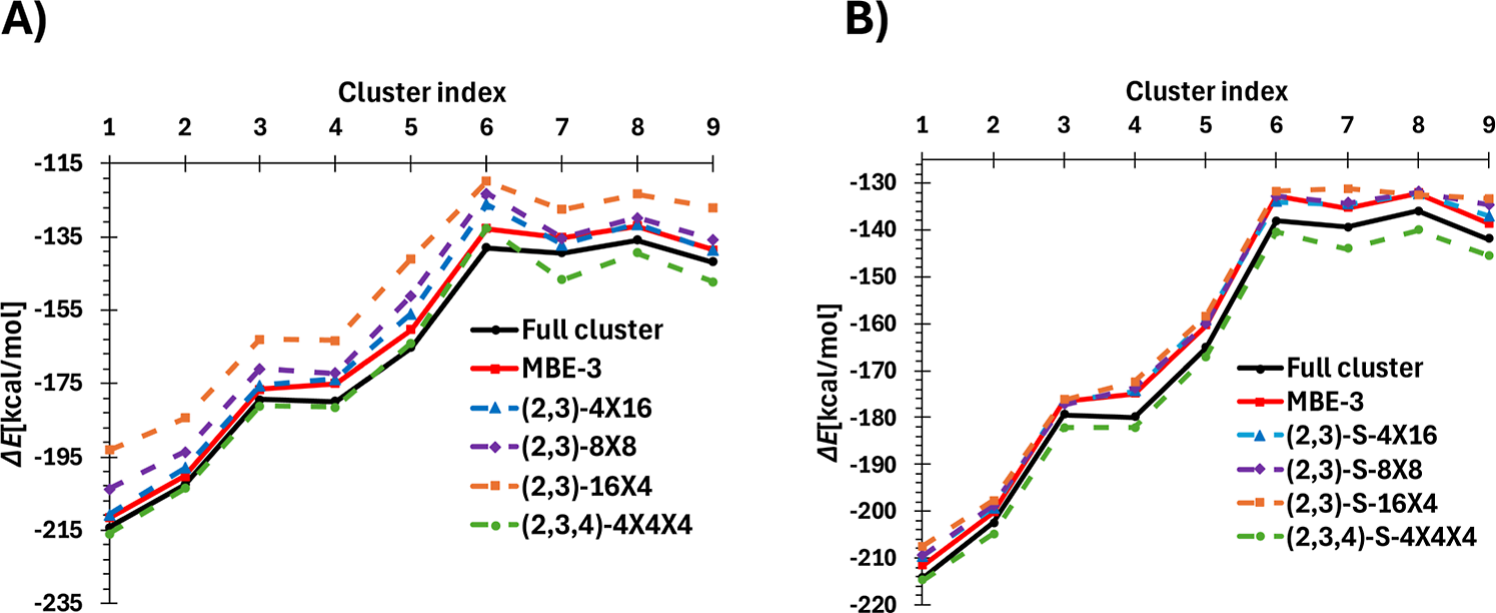
(**A**) Binding energies of the nine (H_2_O)_64_ structures for the regular HMBE partitions (2,3)-4 × 16, (2,3)-8 × 8, (2,3)-16 × 4, and (2,3,4)-4 × 4 × 4. (**B**) Binding energies of the nine (H_2_O)_64_ structures for the Schengen-corrected HMBE partitions (2,3)-S-4 × 16, (2,3)-S-8 × 8, (2,3)-S-16 × 4, (2,3,4)-S-4 × 4 × 4. The reference MBE-3 and full-cluster values are shown for comparison.

The MUE/f of the binding energy as a function of the number of *n*-mers and as a function of the percentage of trimers computed are shown in Figure 5A,B, respectively. Numerical values of the MSE/f and MUE/f are listed in Table S7 of the Supporting Information. Like the relative energies, the MUE/f of the binding energy decreases in the order (2,3)-16 × 4 > (2,3)-8 × 8 > (2,3)-4 × 16, at 0.267, 0.140, and 0.085 kcal/mol, respectively. The addition of Schengen terms further decreases these values to 0.096, 0.076, and 0.069 kcal/mol, respectively. It is emphasized again that these values are comparable to the MBE-3 MUE/f of 0.060 kcal/mol. Interestingly, the MUE/f for the (2,3,4)-4 × 4 × 4 and (2,3,4)-S-4 × 4 × 4 HMBE partitions are both lower than for MBE-3, with values of 0.050 and 0.043 kcal/mol per elementary fragment, respectively. The binding energy is therefore improved when including the 4-body interactions. The number of terms computed for this partition (∼123,000 terms for (2,3,4)-4 × 4 × 4 and ∼132,000 terms for (2,3,4)-S-4 × 4 × 4) is larger than for MBE-3 (∼44,000 terms), but significantly smaller than for MBE-4 (∼635,000 terms). Finally, it is emphasized that while the error in absolute binding energy may seem large (several kcal/mol), the error per fragment is small (in the order of 0.1 kcal/mol) and is expected to hold at larger system sizes for which the method was intended.

**Figure 5:**
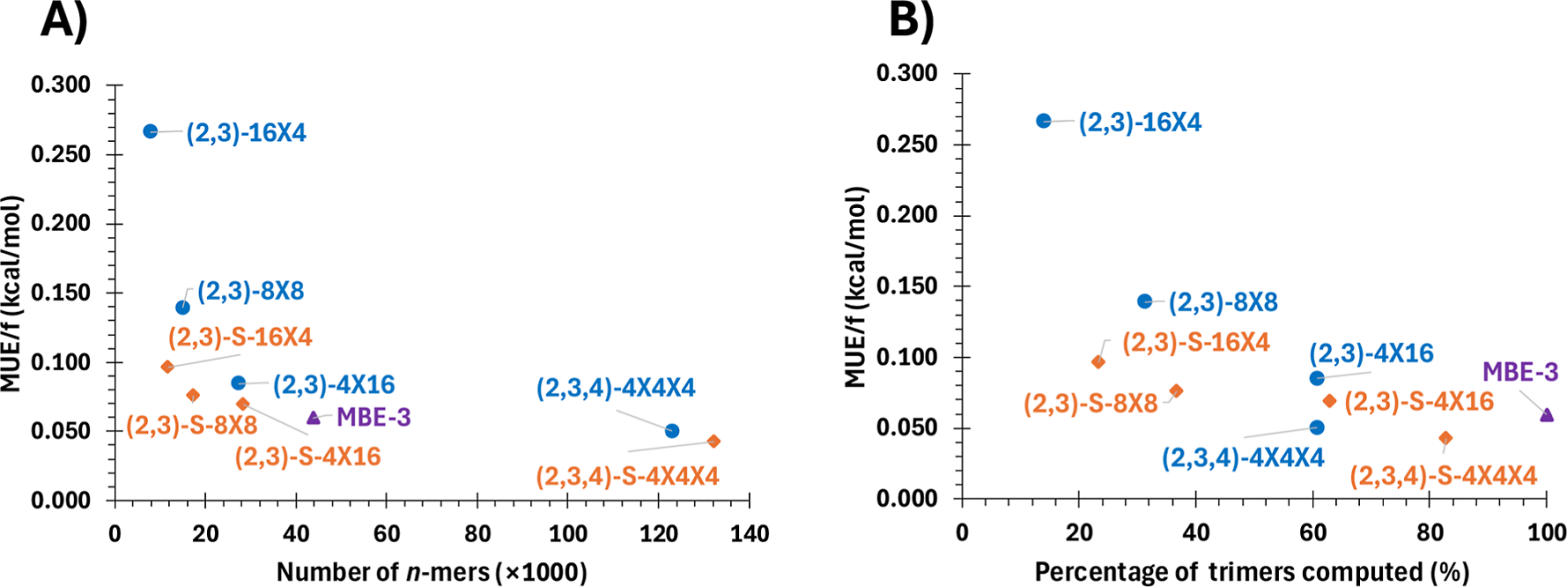
Mean unsigned error per fragment (MUE/f) of the binding energy with respect to full-cluster calculation against (**A**) the total number of terms (*n*-mers) computed and (**B**) the percentage of trimers computed. Regular HMBE calculations are shown by blue circles, Schengen-augmented HMBE calculations by orange diamonds, and conventional MBE-3 by purple triangle.

### 4.3 Scalability of HMBE

The cost-saving advantage of HMBE is expected to grow as the number of monomers increases in a model system. To better illustrate this point, the total number of terms in (*T*_1_,*T*_2_)-HMBE calculations for a series of hypothetical *N*-by-*N* models are listed in Table S8 of the Supporting Information and plotted in Figure 6 against the number of monomers. (Note the logarithm scales in both the *x* and *y* axes.) The number of terms in (*T*_1_,*T*_2_,*T*_3_)-HMBE calculations for a series of hypothetical *N*-by-*N*-by-*N* models is also presented. The number of augmented Schengen terms is not considered here.

**Figure 6:**
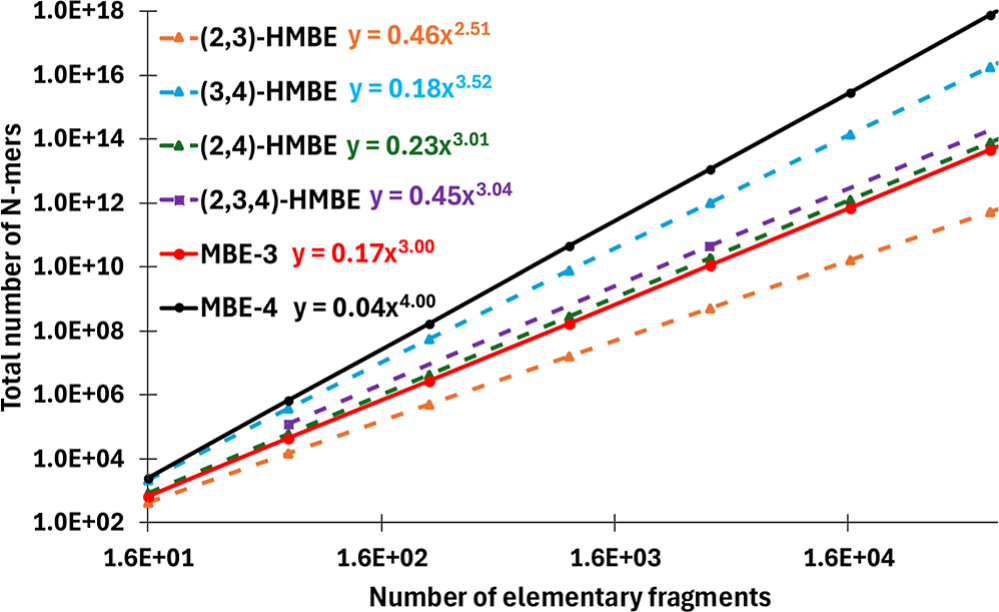
Total number of many-body terms computed for a cluster with (2,3)-HMBE, (2,4)-HMBE, (3,4)-HMBE, (2,3,4)-HMBE, MBE-3 and MBE-4.

The power scaling of (*T*_1_,*T*_2_)-HMBE is evident from the trendlines in Figure 6. It is not surprising that the effective order *n*_eff_, indicated by the *x* exponent of the trendline equation, is lying near the midpoint between *T*_1_ and *T*_2_ due to the symmetric (square *N*-by-*N*) partitions of the monomers. For example, *n*_eff_∼2.5, 3.0 and3.5 for (2,3)-, (2,4)- and (3,4)-HMBE, respectively. As the number of monomers increases, the difference in the number of many-body terms become more substantial, and the saving in hierarchical truncation grows, especially when considering that the neglected terms are all high-order terms that are more expensive to compute. For (2,3,4)-HMBE, the power scaling is *n*_eff_∼3.0, similar to MBE-3.

## 5 Conclusions

In conclusion, a hierarchical many-body expansion (HMBE) method is proposed. HMBE systematically neglects certain terms in the many-body expansion, according to how the fragments are situated in a tiered-structure, which naturally exists in many systems but can also be manually defined. Such a treatment significantly reduces the number of high-order terms to be calculated compared with the conventionally truncated many-body expansion. Two ways to implement HMBE are outlined. The top-down approach moves from the top tier to the bottom tier following the hierarchy of the system. In contrast, the bottom-up approach starts with a conventionally truncated MBE and omits terms that do not satisfy the HMBE truncations.

To further improve the accuracy of HMBE, the hierarchical truncation can be augmented with additional Schengen terms (i.e., the many-body terms at the interface between the fragments) selected using preset cutoff thresholds between atoms. As proof of concept, HMBE is applied to a series of (H_2_O)_64_ extracted from a trajectory generated by a classical MD simulation. The water molecules are hierarchically grouped into three two-tiered partitions: (2,3)-16 × 4, (2,3)-8 × 8, and (2,3)-4 × 16 and one three-tiered partition: (2,3,4)-4 × 4 × 4. The relative energies and total binding energies are in overall good agreement with full cluster calculations and with conventionally truncated MBE-3 calculations. The inclusion of Schengen terms further improves the accuracy of the results due to the increased number of trimers computed. In general, for a (*T*_1_, *T*_2_) partition, the relative energies and binding energies are more accurate when the number of second-tier fragments is larger than the number of first-tier fragments, which is consistent with the fact that more 3-body terms are computed. The three-tiered partition (2,3,4)-4 × 4 × 4 shows relative energies comparable to MBE-3 but with a higher computational cost as 15% of all tetramers are included. On the other hand, the binding energies computed with the three-tier (2,3,4)-4 × 4 × 4 partition are more accurate than those obtained with MBE-3, with a computational cost significantly lower than MBE-4. Overall, the accuracy of the HMBE results also depends on the specific partitioning used for the calculation.

The effective scaling order of (*T*_1_, *T*_2_)-HMBE with respect to the number of elementary fragments, *n*_eff,_ lies between *T*_1_ and *T*_2_. Thus, HMBE scales more favorably than the conventionally truncated MBE that has the same truncation order as *T*_2_, making HMBE a promising choice in MBE applications. This is especially true when HMBE is augmented by Schengen terms to boost its accuracy, because the computational costs of the added Schengen terms are small when the number of second-tier fragments per first tier fragment is larger than the number of first-tier fragments.

Several directions can be further explored to improve the efficiency and accuracy of HMBE. One of them is using higher levels of theory than HF, such as DFT or MP2. While the HF wave function was chosen here to be able to compare results with full cluster calculations, the method can be applied to any other QM level of theory. Another is the optimal selection of truncation orders for different tiers, especially when three or more tiers are present in the system. Another is the development of more efficient and effective Schengen selection criteria to enlist Schengen terms, the inclusion of which markedly enhances the accuracy of HMBE. Furthermore, incorporating electrostatic embedding (as in the EEMB scheme) would likely capture effectively a portion of the critical many-body polarization effects beyond the truncation order at the tier of the elementary fragments, accelerating the convergence of HMBE toward the full MBE. Finally, the HMBE algorithm can be implemented within other fragmentation methods that are built on the many-body expansion^3^ to reduce the number of subsystem computations. With these advancements, HMBE will likely see wider applications in the future.

## Supplementary Material



## Notes

The detailed expression and discussion of the individual terms in eq 13. Discussion of HMBE numerical error propagation. In Table S1, the total number of 1-body, 2-body, 3-body and 4-body terms computed for the (H_2_O)_64_ structures with HMBE and MBE methods. In Table S2, the HMBE, MBE-3 and full cluster absolute energies of the nine (H_2_O)_64_ systems. In Table S3, the Schengen augmented absolute energies of the (H_2_O)_64_ structures. In Table S4, the HMBE, Schengen augmented HMBE, MBE-3 and full cluster relative energies of the nine (H_2_O)_64_ structures. In Table S5, the MUE and MSE of the relative energy per elementary fragment obtained with HMBE, Schengen augmented HMBE, and MBE compared to full cluster methods. In Table S6, the HMBE, Schengen augmented HMBE and MBE total binding energy of the (H_2_O)_64_ structures. In Table S7, the MUE and MSE of the binding energy per elementary fragment obtained with HMBE, Schengen augmented HMBE, and MBE compared to full cluster methods. In Table S8, the number of energy terms computed with the different (2,3), (2,4), (3,4) HMBE truncations for various *N* × *N* partitions, and with (2,3,4) truncation for various *N* × *N* × *N* partitions. In Table S9, the list of Cartesian coordinates and partitions of the nine (H_2_O)_64_ structures (PDF)
