# Supplementary material for: Hierarchical Truncations for Many-Body Expansion Potentials
Source: J Chem Theory Comput. 2026 May 20;22(12):6065–75. doi: 10.1021/acs.jctc.6c00471 (PMC13296478; doi:10.1021/acs.jctc.6c00471)
Supplement: Supplementary file 1 [file ct6c00471_si_001.pdf]

Feb 17, 2025
Supporting Information for

Hierarchical Truncations for Many-body Expansion Potentials

Bryce M. Westheimer[1,2,3,4], Mark S. Gordon[3,4*], Emilie B. Guidez[1,2,*] and Hai Lin[1,2*]

[1]*Department of Chemistry, University of Colorado, Denver, Colorado, 80217, USA*

[2]*Center for Advanced Computational Molecular Sciences, University of Colorado, Denver, Colorado, 80217, USA*

[3]*Ames Laboratory, Ames, Iowa, 50011, USA*

[4]*Department of Chemistry, Iowa State University, Ames, Iowa, 50011, USA*

*Correspondence: mgordon@iastate.edu (M.S.G); emilie.guidez@ucdenver.edu (E.B.G.); hai.lin@ucdenver.edu (H.L.)

Table of Contents

Details of the terms in Eq. 13. 3

Hierarchical Truncations: Numerical Error Propagation. 4

Table S1. Total number of 1-body, 2-body, 3-body and 4-body terms computed for the $(H_2O)_{64}$ clusters with HMBE and MBE methods. The corresponding percentage of trimers and tetramers is given in the last two columns. 6

Table S2. Absolute energies (in Hartree) of the $(H_2O)_{64}$ clusters with HMBE partitions (T_1,T_2)-4X16, (T_1,T_2)-8X8, (T_1,T_2)-16X4 and (T_1,T_2,T_3)-4X4X4, third-order MBE, and at the full HF/aug-cc-pVDZ level. 6

Table S3. Absolute energies (in Hartree) of the $(H_2O)_{64}$ clusters for the Schengen-corrected HMBE calculations: (T_1,T_2)-S-4X16, (T_1,T_2)-S-8X8, (T_1,T_2)-S-16X4 and (T_1,T_2,T_3)-S-4X4X4. 7

Table S4. Relative energies (in kcal/mol) with respect to Cluster 1 for the HMBE, MBE and full cluster calculations. 7

Table S5. Mean signed error (MSE) and mean unsigned error (MUE) of the relative energy per elementary fragment in kcal/mol obtained for the HMBE and MBE-3 calculations relative to the full cluster calculation 7

Table S6. Binding energies (in kcal/mol) of clusters 1-9 for the HMBE, MBE and full cluster calculations. 8

Table S7. Mean signed error (MSE) and mean unsigned error (MUE) of the binding energy per elementary fragment in kcal/mol obtained for the HMBE and MBE-3 calculations relative to the full cluster calculation 8

Table S8. Number of n-body terms in (T_1,T_2)-HMBE and MBE for $N \times N$ fragments and in (T_1,T_2,T_3)-HMBE for $N \times N \times N$ fragments. 9

Table S9. List of the Cartesian coordinates (in Å) and HMBE partitions of $(H_2O)_{64}$ water clusters. The first number after the W is the 1st-tier fragment the water molecule is assigned to, the second number the 2nd tier fragment, and the third number the 3rdtier fragment. 10

Details of the terms in Eq. 13.

The number of 4th order terms in (2,3,4)-HMBE is given by the following sum (**Eq. 13** of the manuscript):

$$\begin{aligned} N_{\text{4th}}^{(2,3,4)-\text{HMBE}}&(M, M_I, M_{Ii}) \\ &= N_{\text{4th}}\left(\text{one } 1^{\text{st}}\text{-tier, one } 2^{\text{nd}}\text{-tier}\right) + N_{\text{4th}}\left(\text{one } 1^{\text{st}}\text{-tier, two } 2^{\text{nd}}\text{-tiers}\right) \\ &+ N_{\text{4th}}\left(\text{one } 1^{\text{st}}\text{-tier, three } 2^{\text{nd}}\text{-tiers}\right) + N_{\text{4th}}\left(\text{two } 1^{\text{st}}\text{-tiers, two } 2^{\text{nd}}\text{-tiers}\right) \\ &+ N_{\text{4th}}\left(\text{two } 1^{\text{st}}\text{-tiers, three } 2^{\text{nd}}\text{-tiers}\right) \end{aligned} \quad (S1)$$

Note that it is again assumed that M_{Ii} has the same value for all 2nd-tier fragments. In other words, all 2nd-tier fragments have the same number of 3rd-tier fragments.

$N_{\text{4th}}\left(\text{one } 1^{\text{st}}\text{-tier, one } 2^{\text{nd}}\text{-tier}\right)$ indicates the number of 4th order terms where the four 3rd-tier elementary fragments belong to the same 1st and 2nd-tier fragments:

$$N_{\text{4th}}\left(\text{one } 1^{\text{st}}\text{-tier, one } 2^{\text{nd}}\text{-tier}\right) = \binom{M}{1}\binom{M_I}{1}\binom{M_{Ii}}{4} \quad (S2)$$

$N_{\text{4th}}\left(\text{one } 1^{\text{st}}\text{-tier, two } 2^{\text{nd}}\text{-tier}\right)$ indicates the number of 4th order terms where the four 3rd-tier elementary fragments belong to *the same* 1st-tier fragment and two *different* 2nd-tier fragments:

$$N_{\text{4th}}\left(\text{one } 1^{\text{st}}\text{-tier, two } 2^{\text{nd}}\text{-tiers}\right) = \binom{M}{1}\binom{M_I}{2}\left(\binom{2}{1}\binom{M_{Ii}}{3}\binom{M_{Ii}}{1} + \binom{M_{Ii}}{2}\binom{M_{Ii}}{2}\right) \quad (S3)$$

$\binom{M_{Ii}}{3}\binom{M_{Ii}}{1}$ indicates that three 3rd-tier elementary fragments are selected from one 2nd-tier fragment while the 4th 3rd-tier elementary fragment is selected from a *different* 2nd-tier fragment. Similarly, $\binom{M_{Ii}}{2}\binom{M_{Ii}}{2}$ indicates that two 3rd-tier elementary fragments are selected from one 2nd-tier fragment while the other two are selected from a different 2nd-tier fragment.

$N_{\text{4th}}\left(\text{one } 1^{\text{st}}\text{-tier, three } 2^{\text{nd}}\text{-tier}\right)$ indicates the number of 4th order terms where the four 3rd-tier elementary fragments belong to the same 1st-tier fragment and three *different* 2nd-tier fragments:

$$N_{\mathrm{4th}}(\text{one } 1^{\mathrm{st}}\text{-tier, three } 2^{\mathrm{nd}}\text{-tiers}) = \binom{M}{1}\binom{M_I}{3}\left(\binom{3}{1}\binom{M_{Ii}}{2}\binom{M_{Ii}}{1}\binom{M_{Ii}}{1}\right) \quad (S4)$$

$\binom{M_{Ii}}{2}\binom{M_{Ii}}{1}\binom{M_{Ii}}{1}$ indicates that two 3rd-tier elementary fragments are selected from the same 2nd-tier fragment while the 3rd and 4th 3rd-tier elementary fragments are selected from two *differen*t 2nd-tier fragments.

$N_{\mathrm{4th}}(\text{two } 1^{\mathrm{st}}\text{-tiers, two } 2^{\mathrm{nd}}\text{-tiers})$ indicates the number of 4th order terms where the four 3rd-tier elementary fragments belong to two different 1st-tier fragments and two different 2nd-tier fragments:

$$N_{\mathrm{4th}}(\text{two } 1^{\mathrm{st}}\text{-tiers, two } 2^{\mathrm{nd}}\text{-tiers}) = \binom{M}{2}\left(\binom{M_I}{1}\binom{M_I}{1}\right)\left(\binom{2}{1}\binom{M_{Ii}}{3}\binom{M_{Ii}}{1} + \binom{M_{Ii}}{2}\binom{M_{Ii}}{2}\right) \quad (S5)$$

$N_{\mathrm{4th}}(\text{two } 1^{\mathrm{st}}\text{-tiers, three } 2^{\mathrm{nd}}\text{-tiers})$ indicates the number of 4th order terms where the four 3rd-tier elementary fragments belong to two different 1st-tier fragments and three different 2nd-tier fragments:

$$N_{\mathrm{4th}}(\text{two } 1^{\mathrm{st}}\text{-tiers, three } 2^{\mathrm{nd}}\text{-tiers}) = \binom{M}{2}\left(\binom{2}{1}\binom{M_I}{1}\binom{M_I}{2}\right)\left(\binom{3}{1}\binom{M_{Ii}}{2}\binom{M_{Ii}}{1}\binom{M_{Ii}}{1}\right) \quad (S6)$$

Hierarchical Truncations: Numerical Error Propagation.

For conventional MBE truncated at the n-order, the numerical errors accumulated when the individual terms are summed is given by:

$$\delta E^{MBE}(m,n) = \delta E_X \left(\sum_{t=0}^{n-1}\binom{m}{n-t}\left(\frac{(m-n-1+t)!}{t!\,(m-n-1)!}\right)^2\right)^{1/2} \quad (S7)$$

where the numerical error δE_X is assumed to be the same for each fragment X. The numerical errors arise from finite precisions in numerical calculations. For example, a Hartree-Fock energy with an energy convergence threshold of 10^{-6} Hartree has an uncertainty δE of 10^{-6} Hartree for every computation. Although the uncertainties may be small individually, due to the large number of terms

involved, the accumulation of these errors can be of concern. Of course, it is possible to tighten convergence thresholds to reduce numerical uncertainty, at the cost of more SCF cycles and longer computational times.

Similar detailed analysis can be performed for HMBE, but it is rather cumbersome and not pursued here. Because of the omitted terms, one would expect that HMBE generally possesses smaller accumulated numerical errors than conventionally truncated MBE. Applying Stirling's approximation to **Eq. 16**, it is easy to show that $\delta E^{\mathrm{MBE}}(m,n)$ scales as $m^{n-0.5}$ when $m \gg n$. Therefore, it is not unreasonable to expect that the accumulated numerical error in $(T_1, T_2, \ldots, T_{\mathrm{max}})$-HMBE scales as $m^{n_{\mathrm{eff}}-0.5}$ if $m \gg T_{\mathrm{max}}$, where T_{max} is the truncation order at the lowest-tier for the elementary fragments.

Table S1. Total number of 1-body, 2-body, 3-body and 4-body terms computed for the $(H_2O)_{64}$ clusters with HMBE and MBE methods. The corresponding percentage of trimers and tetramers is given in the last two columns.

Model	1-body	2-body	3-body	4-body	Total	%trimers	%tetramers
(2,3)-4X16	64	2016	25280	0	27360	60.7	0
(2,3)-8X8	64	2016	12992	0	15072	31.2	0
(2,3)-16X4	64	2016	5824	0	7904	14.0	0
(2,3,4)-4X4X4	64	2016	25280	95728	123088	60.7	15.1
(2,3)-S-4X16	64	2016	26203	0	28283[a]	62.9	0
(2,3)-S-8X8	64	2016	15332	0	17412[a]	36.8	0
(2,3)-S-16X4	64	2016	9694	0	11774[a]	23.3	0
(2,3,4)-S-4X4X4	64	2016	34513	95728	132321[a]	82.8	15.1
MBE-2	64	2016	0	0	2080	0	0
MBE-3	64	2016	41664	0	43744	100	0
MBE-4	64	2016	41664	635376	679120	100	100

a. The number of Schengen terms is averaged over all 9 clusters.

Table S2. Absolute energies (in Hartree) of the $(H_2O)_{64}$ clusters with HMBE partitions (T_1,T_2)-4X16, (T_1,T_2)-8X8, (T_1,T_2)-16X4 and (T_1,T_2,T_3)-4X4X4, third-order MBE, and at the full HF/aug-cc-pVDZ level.

Cluster	(2,3)-4X16	(2,3)-8X8	(2,3)-16X4	(2,3,4)-4X4X4	MBE-3	Full cluster
1	-4866.9873622343	-4866.9763227962	-4866.9593373617	-4866.9955600176	-4866.9886090000	-4866.9929096623
2	-4866.9670754571	-4866.9600043441	-4866.9452850289	-4866.9759578560	-4866.9705730000	-4866.9741477491
3	-4866.9311146032	-4866.9238901618	-4866.9112499778	-4866.9401235483	-4866.9326817792	-4866.9372894661
4	-4866.9282379363	-4866.9258037944	-4866.9117929782	-4866.9406057293	-4866.9302061534	-4866.9381015632
5	-4866.9000620095	-4866.8923247787	-4866.8761845614	-4866.9127413483	-4866.9069394542	-4866.9146874320
6	-4866.8524159750	-4866.8478335467	-4866.8425743938	-4866.8627344736	-4866.8629855871	-4866.8714800542
7	-4866.8700499328	-4866.8671728095	-4866.8545833384	-4866.8852286711	-4866.8670364085	-4866.8734743665
8	-4866.8611915178	-4866.8583240355	-4866.8479860463	-4866.8737071416	-4866.8619311202	-4866.8681302410
9	-4866.8723571235	-4866.8678596037	-4866.8539275386	-4866.8859546644	-4866.8722109760	-4866.8775983687

Table S3. Absolute energies (in Hartree) of the $(H_2O)_{64}$ clusters for the Schengen-corrected HMBE calculations: (T_1,T_2)-S-4X16, (T_1,T_2)-S-8X8, (T_1,T_2)-S-16X4 and (T_1,T_2,T_3)-S-4X4X4.

Cluster	(2,3)-S-4X16	(2,3)-S-8X8	(2,3)-S-16X4	(2,3,4)-S-4X4X4
1	-4866.9855192343	-4866.9852967962	-4866.9822173617	-4866.9937210176
2	-4866.9691124571	-4866.9681833441	-4866.9665990289	-4866.9779988560
3	-4866.9326576032	-4866.9339781618	-4866.9323279778	-4866.9416715483
4	-4866.9293409363	-4866.9281877944	-4866.9262199782	-4866.9417117293
5	-4866.9050920095	-4866.9065417787	-4866.9039435614	-4866.9177763483
6	-4866.8649089750	-4866.8630765467	-4866.8614433938	-4866.8752344736
7	-4866.8658229328	-4866.8653508095	-4866.8604723384	-4866.8810056711
8	-4866.8621735178	-4866.8616330355	-4866.8625120463	-4866.8746951416
9	-4866.8696811235	-4866.8659976037	-4866.8638425386	-4866.8832836644

Table S4. Relative energies (in kcal/mol) with respect to Cluster 1 for the HMBE, MBE and full cluster calculations.

Model	1	2	3	4	5	6	7	8	9
(2,3)-4X16	0	12.73	35.30	37.10	54.78	84.68	73.61	79.17	72.17
(2,3)-8X8	0	10.24	32.90	31.70	52.71	80.63	68.49	74.05	68.06
(2,3)-16X4	0	8.82	30.18	29.83	52.18	73.27	65.73	69.87	66.15
(2,3,4)-4X4X4	0	12.30	34.79	34.48	51.97	83.35	69.23	76.46	68.78
(2,3)-S-4X16	0	10.30	33.17	35.25	50.47	75.68	75.11	77.40	72.69
(2,3)-S-8X8	0	10.74	32.20	35.84	49.42	76.69	75.27	77.60	74.86
(2,3)-S-16X4	0	9.80	31.31	35.14	49.12	75.79	76.40	75.12	74.28
(2,3,4)-S-4X4X4	0	9.87	32.66	32.64	47.66	74.35	70.73	74.69	69.30
MBE-3	0	11.32	35.09	36.65	51.25	78.83	76.29	79.49	73.04
Full cluster	0	11.77	34.90	34.39	49.09	76.20	74.95	78.30	72.36

Table S5. Mean signed error (MSE) and mean unsigned error (MUE) of the relative energy per elementary fragment in kcal/mol obtained for the HMBE and MBE-3 calculations relative to the full cluster calculation.

Model	MSE	MUE
(2,3)-4X16	0.031	0.036
(2,3)-8X8	-0.023	0.051
(2,3)-16X4	-0.062	0.073
(2,3,4)-4X4X4	-0.001	0.038
(2,3)-S-4X16	-0.003	0.013
(2,3)-S-8X8	0.001	0.017
(2,3)-S-16X4	-0.009	0.023
(2,3,4)-S-4X4X4	-0.035	0.035
MBE-3	0.017	0.019

Table S6. Binding energies (in kcal/mol) of clusters 1-9 for the HMBE, MBE and full cluster calculations.

Model	1	2	3	4	5	6	7	8	9
(2,3)-4X16	-210.82	-198.09	-175.53	-173.73	-156.04	-126.14	-137.21	-131.65	-138.67
(2,3)-8X8	-203.89	-193.66	-171.00	-172.20	-151.18	-123.26	-135.41	-129.85	-135.84
(2,3)-16X4	-193.24	-184.42	-163.06	-163.41	-141.05	-119.96	-127.51	-123.37	-127.10
(2,3,4)-4X4X4	-215.97	-203.67	-181.18	-181.49	-163.99	-132.61	-146.74	-139.51	-147.20
(2,3)-S-4X16	-209.67	-199.37	-176.50	-174.42	-159.19	-133.98	-134.56	-132.27	-136.99
(2,3)-S-8X8	-209.53	-198.79	-177.33	-173.70	-160.10	-132.83	-134.26	-131.93	-134.68
(2,3)-S-16X4	-207.59	-197.79	-176.29	-172.46	-158.47	-131.80	-131.20	-132.48	-133.32
(2,3,4)-S-4X4X4	-214.81	-204.95	-182.15	-182.18	-167.15	-140.46	-144.09	-140.13	-145.52
MBE-3	-211.60	-200.29	-176.51	-174.96	-160.35	-132.77	-135.32	-132.12	-138.57
Full cluster	-214.30	-202.53	-179.40	-179.92	-165.22	-138.10	-139.36	-136.01	-141.96

Table S7. Mean signed error (MSE) and mean unsigned error (MUE) of the binding energy per elementary fragment in kcal/mol obtained for the HMBE and MBE-3 calculations relative to the full cluster calculation.

Model	MSE	MUE
(2,3)-4X16	0.085	0.085
(2,3)-8X8	0.140	0.140
(2,3)-16X4	0.267	0.267
(2,3,4)-4X4X4	-0.027	0.050
(2,3)-S-4X16	0.069	0.069
(2,3)-S-8X8	0.076	0.076
(2,3)-S-16X4	0.096	0.096
(2,3,4)-S-4X4X4	-0.043	0.043
MBE-3	0.060	0.060

Table S8. Number of *n*-body terms in (T_1,T_2)-HMBE and MBE for *N*×*N* fragments and in (T_1,T_2,T_3)-HMBE for *N*×*N*×*N* fragments.

N	**1-body**	**2-body**	**3-body**	**4-body**	**Total**
**(2,3)-HMBE**					
4	16	120	304	0	440
8	64	2016	12992	0	15072
16	256	32640	469760	0	502656
32	1024	523776	15903744	0	16428544
64	4096	8386560	5.23E+08	0	5.31E+08
128	16384	1.34E+08	1.70E+10	0	1.71E+10
**(2,4)-HMBE**					
4	16	120	304	412	852
8	64	2016	12992	47600	62672
16	256	32640	469760	3907520	4410176
32	1024	523776	15903744	2.81E+08	2.97E+08
64	4096	8386560	5.23E+08	1.90E+10	1.95E+10
128	16384	1.34E+08	1.70E+10	1.25E+12	1.27E+12
**(3,4)-HMBE**					
4	16	120	560	1564	2260
8	64	2016	41664	348656	392400
16	256	32640	2763520	55517120	58313536
32	1024	523776	1.78E+08	7.84E+09	8.02E+09
64	4096	8386560	1.14E+10	1.05E+12	1.06E+12
128	16384	1.34E+08	7.33E+11	1.38E+14	1.38E+14
**(2,3,4)-HMBE**					
4	64	2016	25280	95728	123088
16	4096	838650	2.05E+09	4.43E+10	4.64E+10
64	262144	3.44E+10	1.39E+14	1.30E+16	1.32E+16
256	16777216	1.41E+14	9.20E+18	3.51E+21	3.52E+21
**MBE-3**					
4	16	120	560	0	696
8	64	2016	41664	0	43744
16	256	32640	2763520	0	2796416
32	1024	523776	1.78E+08	0	178957824
64	4096	8386560	1.14E+10	0	1.15E+10
128	16384	1.34E+08	7.33E+11	0	7.33E+13
**MBE-4**					
4	16	120	560	1820	2516
8	64	2016	41664	635376	679120
16	256	32640	2763520	1.75E+08	1.78E+08
32	1024	523776	1.78E+08	4.55E+10	4.57E+10
64	4096	8386560	1.14E+10	1.17E+13	1.17E+13
128	16384	1.34E+08	7.33E+11	3.00E+15	3.00E+15

Table S9. List of the Cartesian coordinates (in Å) and HMBE partitions of $(H_2O)_{64}$ water clusters. The first number after the W is the 1st-tier fragment the water molecule is assigned to, the second number the 2nd tier fragment, and the third number the 3rd tier fragment.

Cluster 1

Element	X	Y	Z	hmbe_4_16	hmbe_16_4	hmbe_8_8	hmbe_4_4_4
O	3.276	-2.575	-4.013	W1_1	W12_2	W1_3	W1_1_4
H	3.619	-3.239	-3.416				
H	2.381	-2.858	-4.2				
O	3.567	-1.726	5.939	W1_2	W8_2	W7_5	W1_2_1
H	3.366	-2.154	5.106				
H	2.711	-1.511	6.31				
O	4.333	-2.861	-0.207	W1_3	W14_4	W1_2	W1_2_2
H	4.976	-2.156	-0.277				
H	4.529	-3.282	0.63				
O	3.196	-3.379	3.601	W1_4	W8_3	W6_2	W1_3_4
H	2.393	-2.904	3.387				
H	3.066	-4.25	3.225				
O	7.092	1.257	-1.434	W1_5	W5_3	W7_4	W1_4_3
H	7.117	0.325	-1.648				
H	7.585	1.329	-0.617				
O	2.677	-6.752	-3.086	W1_6	W12_3	W1_1	W1_1_3
H	3.359	-6.139	-2.812				
H	2.537	-7.312	-2.323				
O	2.501	1.471	-4.204	W1_7	W15_3	W3_8	W1_4_2
H	1.702	0.948	-4.268				
H	2.424	1.924	-3.364				
O	6.759	-1.414	-3.689	W1_8	W5_1	W1_5	W1_4_1
H	7.068	-2.041	-4.343				
H	6.02	-0.974	-4.109				
O	6.23	0.486	2.977	W1_9	W9_3	W7_1	W1_2_3
H	7.009	-0.029	2.768				
H	5.894	0.096	3.785				
O	4.827	-6.035	1.733	W1_10	W14_3	W1_4	W1_3_3
H	3.968	-5.994	1.312				
H	4.635	-6.016	2.671				
O	1.399	-2.596	-0.081	W1_11	W14_2	W1_6	W1_3_2
H	2.285	-2.519	-0.436				
H	0.991	-3.289	-0.601				
O	5.587	-2.602	2.542	W1_12	W14_1	W7_8	W1_2_4

H	5.839	-1.813	3.021				
H	4.827	-2.939	3.017				
O	4.508	-0.312	-4.79	W1_13	W5_4	W3_2	W1_4_4
H	4.035	-1.078	-4.463				
H	3.912	0.421	-4.637				
O	4.542	-4.639	-2.197	W1_14	W5_2	W1_7	W1_1_1
H	4.665	-3.963	-1.53				
H	5.379	-5.103	-2.224				
O	0.817	-3.905	-3.703	W1_15	W12_4	W1_8	W1_1_2
H	0.263	-4.62	-3.388				
H	1.13	-4.202	-4.557				
O	2.099	-5.361	2.055	W1_16	W4_4	W6_8	W1_3_1
H	1.476	-5.157	1.357				
H	1.651	-6.014	2.593				
O	-6.188	1.371	1.124	W2_1	W10_2	W8_3	W2_3_4
H	-5.595	0.62	1.15				
H	-6.931	1.072	0.6				
O	-1.569	1.601	-6.573	W2_2	W7_2	W3_5	W2_4_2
H	-1.37	1.442	-7.495				
H	-2.485	1.338	-6.476				
O	-0.992	2.01	-2.774	W2_3	W15_1	W3_3	W2_2_3
H	-0.589	2.112	-3.637				
H	-1.926	1.91	-2.957				
O	-7.235	-1.615	-3.611	W2_4	W10_4	W8_5	W2_1_1
H	-6.299	-1.568	-3.418				
H	-7.433	-0.786	-4.046				
O	-2.878	-1.352	-1.883	W2_5	W6_2	W4_3	W2_1_2
H	-2.556	-1.491	-0.992				
H	-3.702	-1.838	-1.918				
O	-2.579	5.357	-3.45	W2_6	W16_4	W2_4	W2_2_4
H	-2.24	6.196	-3.764				
H	-1.886	4.728	-3.653				
O	-3.352	-1.459	-5.864	W2_7	W7_1	W4_8	W2_4_1
H	-2.631	-1.558	-5.242				
H	-3.594	-0.535	-5.805				
O	-7.439	4.057	-1.004	W2_8	W10_3	W8_4	W2_3_3
H	-7.603	4.603	-1.773				
H	-6.583	3.662	-1.168				
O	0.397	0.118	-5.024	W2_9	W7_4	W3_6	W2_4_4
H	-0.035	-0.705	-4.801				
H	-0.063	0.427	-5.805				

O	-3.926	5.286	-1	W2_10	W3_4	W2_3	W2_2_2
H	-3.963	4.342	-0.844				
H	-3.547	5.37	-1.876				
O	-3.548	0.967	-3.18	W2_11	W3_3	W8_6	W2_1_4
H	-3.572	0.095	-2.786				
H	-4.417	1.08	-3.566				
O	-0.87	3.813	-5.008	W2_12	W16_1	W3_1	W2_2_1
H	-1.114	2.964	-5.376				
H	-0.745	4.378	-5.771				
O	-4.233	-3.947	-5.189	W2_13	W1_1	W4_1	W2_1_3
H	-3.65	-3.226	-5.426				
H	-5.046	-3.763	-5.66				
O	-6.239	1.176	-3.521	W2_14	W10_1	W8_1	W2_3_1
H	-6.448	2.026	-3.907				
H	-6.773	1.135	-2.728				
O	-4.896	2.698	-1.049	W2_15	W3_1	W8_7	W2_3_2
H	-4.436	2.003	-1.52				
H	-5.162	2.293	-0.223				
O	-1.301	-1.979	-4.281	W2_16	W7_3	W4_4	W2_4_3
H	-0.49	-2.435	-4.059				
H	-1.651	-1.686	-3.44				
O	-0.817	-5.701	-2.323	W3_1	W12_1	W4_7	W3_1_4
H	-0.713	-5.685	-1.372				
H	-0.591	-6.596	-2.575				
O	-2.743	-1.325	3.094	W3_2	W11_2	W5_5	W3_2_3
H	-3.085	-0.476	3.373				
H	-2.922	-1.91	3.83				
O	-4.611	-3.925	0.876	W3_3	W6_4	W8_8	W3_4_2
H	-5.556	-3.798	0.957				
H	-4.516	-4.821	0.551				
O	0.223	-6.857	3.727	W3_4	W4_1	W6_1	W3_3_1
H	-0.547	-7.25	4.139				
H	0.185	-5.934	3.976				
O	0.584	-2.478	2.682	W3_5	W8_4	W6_4	W3_2_2
H	0.808	-2.461	1.752				
H	0.337	-1.576	2.887				
O	-2.633	-4.83	4.337	W3_6	W11_1	W6_7	W3_3_3
H	-1.711	-4.629	4.495				
H	-2.617	-5.584	3.748				
O	-4.877	-3.205	-2.343	W3_7	W1_3	W4_6	W3_4_1
H	-4.743	-3.531	-3.233				

H	-4.897	-3.992	-1.799				
O	-2.357	-6.751	2.013	W3_8	W4_3	W6_5	W3_3_2
H	-3.114	-7.187	1.623				
H	-2.088	-7.329	2.727				
O	-3.558	-6.418	-1.095	W3_9	W1_2	W4_2	W3_1_2
H	-2.642	-6.68	-0.996				
H	-4.026	-6.926	-0.432				
O	-2.109	-2.316	0.541	W3_10	W6_3	W5_6	W3_4_3
H	-2.169	-1.975	1.434				
H	-2.821	-2.954	0.48				
O	-0.416	-5.269	0.448	W3_11	W4_2	W6_3	W3_1_3
H	-0.867	-4.45	0.653				
H	-0.732	-5.888	1.106				
O	-2.627	-6.022	-4.295	W3_12	W1_4	W4_5	W3_1_1
H	-2.104	-5.726	-3.55				
H	-3.247	-5.309	-4.451				
O	0.045	-4.243	4.461	W3_13	W8_1	W6_6	W3_3_4
H	0.157	-3.572	3.788				
H	0.68	-4.013	5.139				
O	-5.885	-0.86	4.058	W3_14	W11_3	W5_1	W3_2_1
H	-5.233	-1.375	4.532				
H	-5.474	-0.004	3.937				
O	-3.818	-2.49	5.222	W3_15	W11_4	W5_7	W3_2_4
H	-3.95	-2.361	6.161				
H	-3.334	-3.313	5.158				
O	-5.338	-1.217	0.614	W3_16	W6_1	W8_2	W3_4_4
H	-5.133	-1.799	1.346				
H	-5.154	-1.74	-0.167				
O	0.034	5.162	-0.362	W4_1	W16_3	W2_6	W4_2_1
H	0.602	4.487	0.008				
H	0.203	5.128	-1.304				
O	-0.409	4.432	3.76	W4_2	W13_2	W2_7	W4_1_2
H	0.029	5.282	3.806				
H	-1.339	4.645	3.675				
O	-2.191	2.431	2.451	W4_3	W13_3	W2_2	W4_1_3
H	-2.31	2.474	1.502				
H	-2.485	3.285	2.768				
O	4.296	2.567	2.218	W4_4	W9_2	W7_3	W4_4_1
H	4.781	1.803	2.53				
H	3.479	2.553	2.716				
O	0	0	0	W4_5	W15_2	W5_2	W4_3_2

H	0.469	-0.832	-0.057				
H	-0.882	-0.2	-0.313				
O	-0.872	1.503	5.241	W4_6	W2_1	W5_3	W4_3_1
H	-0.614	0.846	4.595				
H	-1.087	2.278	4.721				
O	3.436	4.196	-0.032	W4_7	W9_4	W7_7	W4_4_4
H	3.812	3.641	0.651				
H	2.606	3.776	-0.253				
O	-1.923	6.598	0.641	W4_8	W13_1	W2_1	W4_1_1
H	-1.204	6.03	0.365				
H	-2.655	6.352	0.076				
O	2.554	5.742	3.481	W4_9	W9_1	W7_2	W4_4_2
H	2.42	4.955	2.952				
H	3.144	6.285	2.958				
O	-2.03	2.831	-0.22	W4_10	W3_2	W2_8	W4_2_4
H	-1.586	2.41	-0.956				
H	-1.444	3.542	0.038				
O	0.939	4.506	-2.887	W4_11	W16_2	W3_7	W4_2_3
H	0.412	4.129	-3.591				
H	1.732	4.819	-3.322				
O	-2.861	5.2	3.002	W4_12	W13_4	W2_5	W4_1_4
H	-2.479	5.859	2.423				
H	-3.735	5.044	2.645				
O	-0.368	0.579	2.65	W4_13	W2_3	W5_8	W4_3_4
H	-0.296	0.313	1.733				
H	-0.951	1.338	2.634				
O	-3.672	0.937	3.95	W4_14	W2_2	W5_4	W4_3_3
H	-3.766	1.381	4.793				
H	-3.234	1.577	3.389				
O	0.901	2.571	-0.584	W4_15	W15_4	W3_4	W4_2_2
H	0.866	1.655	-0.308				
H	0.528	2.571	-1.465				
O	1.683	2.469	3.849	W4_16	W2_4	W7_6	W4_4_3
H	1.267	3.289	4.114				
H	1.01	1.803	3.989				

Cluster 2

Element	X	Y	Z	hmbe_4_16	hmbe_16_4	hmbe_8_8	hmbe_4_4_4
O	3.585	-2.455	-1.561	W1_1	W13_1	W3_8	W1_4_1
H	2.943	-3.138	-1.755				

H	4.414	-2.926	-1.467				
O	1.799	-2.406	5.214	W1_2	W9_3	W8_8	W1_2_4
H	2.303	-2.492	4.405				
H	0.887	-2.382	4.927				
O	-1.047	-2.934	2.273	W1_3	W3_3	W5_8	W1_1_2
H	-0.553	-2.354	2.851				
H	-1.964	-2.754	2.479				
O	0.877	-5.384	4.522	W1_4	W6_1	W3_1	W1_2_1
H	0.626	-5.146	3.629				
H	0.211	-4.975	5.075				
O	2.775	-6.890	-0.483	W1_5	W1_2	W3_5	W1_3_2
H	2.361	-7.597	-0.978				
H	2.544	-6.094	-0.961				
O	0.000	0.000	0.000	W1_6	W4_2	W7_2	W1_4_2
H	-0.900	-0.264	0.191				
H	0.092	-0.127	-0.944				
O	5.596	-2.864	2.561	W1_7	W6_4	W8_5	W1_2_3
H	5.492	-3.346	1.740				
H	4.707	-2.780	2.904				
O	-0.799	-1.047	4.090	W1_8	W9_4	W5_3	W1_1_4
H	-0.345	-0.468	4.702				
H	-1.729	-0.879	4.245				
O	2.018	-5.101	1.832	W1_9	W6_3	W3_6	W1_3_1
H	2.302	-5.928	1.442				
H	1.220	-4.873	1.355				
O	2.451	-1.324	0.644	W1_10	W13_2	W3_4	W1_4_3
H	2.759	-1.644	-0.204				
H	1.563	-1.010	0.474				
O	0.070	-3.765	-0.092	W1_11	W4_3	W3_2	W1_3_3
H	-0.748	-4.102	-0.457				
H	-0.206	-3.170	0.605				
O	-0.963	-3.537	5.428	W1_12	W9_1	W5_7	W1_1_3
H	-1.392	-2.887	4.871				
H	-1.185	-3.272	6.321				
O	5.343	-2.335	-3.833	W1_13	W13_4	W4_8	W1_4_4
H	4.736	-2.296	-3.094				
H	6.035	-1.714	-3.609				
O	1.079	0.416	5.438	W1_14	W16_3	W8_6	W1_1_1
H	1.696	-0.313	5.493				
H	1.276	0.834	4.599				
O	2.006	-4.686	-1.844	W1_15	W1_4	W3_3	W1_3_4

H	1.356	-4.283	-1.268				
H	1.527	-4.879	-2.650				
O	2.609	-2.675	2.890	W1_16	W6_2	W8_2	W1_2_2
H	2.427	-3.534	2.508				
H	2.493	-2.061	2.166				
O	-2.670	1.834	4.777	W2_1	W9_2	W5_1	W2_4_2
H	-2.938	1.399	5.587				
H	-1.769	2.112	4.941				
O	-4.656	1.089	0.825	W2_2	W14_3	W6_6	W2_2_2
H	-4.978	1.695	0.158				
H	-5.186	1.281	1.599				
O	-3.815	-2.427	2.120	W2_3	W3_4	W5_2	W2_2_1
H	-4.683	-2.814	2.006				
H	-3.777	-1.726	1.470				
O	-3.090	3.671	-1.578	W2_4	W14_4	W7_8	W2_3_4
H	-2.494	3.850	-0.851				
H	-3.963	3.713	-1.188				
O	-7.141	0.424	-3.941	W2_5	W8_2	W1_7	W2_3_2
H	-8.044	0.617	-3.686				
H	-6.612	0.745	-3.211				
O	-2.677	5.292	2.345	W2_6	W2_2	W6_7	W2_4_1
H	-3.087	4.707	2.983				
H	-2.902	6.172	2.648				
O	-6.327	4.172	2.138	W2_7	W10_2	W6_5	W2_4_3
H	-6.009	3.512	2.754				
H	-7.089	4.557	2.571				
O	-5.457	0.524	5.214	W2_8	W10_1	W6_1	W2_1_4
H	-5.165	1.070	5.944				
H	-6.188	0.019	5.570				
O	-5.549	3.382	-0.436	W2_9	W14_1	W6_2	W2_3_3
H	-6.378	3.487	-0.903				
H	-5.686	3.829	0.400				
O	-2.797	-0.665	-0.331	W2_10	W4_4	W5_4	W2_2_4
H	-3.204	0.066	0.135				
H	-2.927	-0.461	-1.257				
O	-1.517	1.842	2.371	W2_11	W4_1	W5_5	W2_2_3
H	-2.091	1.341	2.951				
H	-1.589	1.401	1.524				
O	-6.653	-2.286	1.745	W2_12	W3_2	W6_4	W2_1_3
H	-6.607	-1.794	0.925				
H	-7.184	-1.739	2.324				

O	-3.518	-1.260	4.505	W2_13	W3_1	W5_6	W2_1_2
H	-4.257	-0.825	4.930				
H	-3.907	-1.730	3.768				
O	-4.394	3.861	4.165	W2_14	W10_3	W6_3	W2_4_4
H	-4.248	4.515	4.848				
H	-3.691	3.223	4.289				
O	-6.431	1.445	3.007	W2_15	W10_4	W6_8	W2_1_1
H	-7.278	1.007	2.927				
H	-6.202	1.360	3.932				
O	-3.364	5.802	-2.888	W2_16	W14_2	W7_5	W2_3_1
H	-2.494	6.029	-3.217				
H	-3.269	4.910	-2.554				
O	1.881	0.129	-6.277	W3_1	W5_2	W4_6	W3_3_2
H	2.830	0.065	-6.167				
H	1.537	-0.655	-5.849				
O	-2.842	-3.401	-1.848	W3_2	W11_1	W1_8	W3_2_4
H	-2.929	-2.964	-1.001				
H	-2.512	-2.722	-2.436				
O	-2.007	1.839	-5.027	W3_3	W12_4	W7_3	W3_1_1
H	-2.548	2.629	-5.025				
H	-1.190	2.106	-4.606				
O	1.167	-5.174	-4.289	W3_4	W1_1	W3_7	W3_3_1
H	1.597	-5.880	-4.772				
H	0.254	-5.212	-4.573				
O	-6.056	-2.222	-3.923	W3_5	W8_4	W1_4	W3_2_1
H	-6.391	-1.444	-3.479				
H	-5.106	-2.106	-3.926				
O	-1.538	-2.680	-6.066	W3_6	W11_2	W1_6	W3_4_3
H	-0.709	-2.510	-6.512				
H	-1.930	-3.403	-6.556				
O	0.301	-0.222	-2.918	W3_7	W11_3	W7_7	W3_1_3
H	1.236	-0.427	-2.887				
H	0.265	0.688	-3.213				
O	2.815	3.417	-6.885	W3_8	W5_3	W4_5	W3_1_2
H	3.379	3.153	-7.612				
H	2.902	2.710	-6.245				
O	-3.634	-5.747	-3.373	W3_9	W8_1	W1_1	W3_2_2
H	-4.497	-6.030	-3.072				
H	-3.593	-4.818	-3.146				
O	-3.987	0.454	-2.785	W3_10	W8_3	W1_2	W3_2_3
H	-3.614	1.327	-2.905				

H	-3.471	-0.109	-3.362				
O	0.568	2.340	-3.962	W3_11	W12_2	W7_6	W3_1_4
H	1.254	2.578	-4.585				
H	0.665	2.968	-3.246				
O	-3.441	-0.300	-6.405	W3_12	W12_1	W1_5	W3_4_1
H	-2.786	-0.992	-6.313				
H	-2.992	0.495	-6.120				
O	0.058	1.325	-8.090	W3_13	W12_3	W4_1	W3_4_2
H	-0.528	1.196	-7.344				
H	0.905	0.995	-7.790				
O	-2.060	-1.466	-3.556	W3_14	W11_4	W1_3	W3_4_4
H	-1.263	-0.994	-3.318				
H	-1.848	-1.890	-4.388				
O	1.345	-2.671	-6.004	W3_15	W1_3	W4_7	W3_3_3
H	1.542	-3.392	-5.405				
H	2.164	-2.522	-6.477				
O	3.987	-1.912	-6.071	W3_16	W5_4	W4_2	W3_3_4
H	4.538	-1.140	-6.201				
H	4.226	-2.233	-5.202				
O	-0.359	3.816	3.850	W4_1	W2_3	W2_3	W4_1_1
H	0.348	4.414	3.607				
H	-0.624	3.410	3.024				
O	3.186	3.688	-0.594	W4_2	W15_4	W2_4	W4_2_4
H	3.284	4.021	-1.485				
H	2.240	3.656	-0.452				
O	4.262	0.767	5.051	W4_3	W16_1	W8_7	W4_3_4
H	4.360	0.108	4.364				
H	4.439	1.599	4.613				
O	3.043	0.112	-2.606	W4_4	W13_3	W2_2	W4_4_2
H	3.946	-0.174	-2.464				
H	2.730	0.359	-1.736				
O	6.940	1.575	3.332	W4_5	W7_2	W8_4	W4_3_2
H	6.042	1.500	3.009				
H	7.455	1.797	2.556				
O	1.502	1.258	2.863	W4_6	W16_2	W8_3	W4_1_2
H	1.112	0.557	2.341				
H	1.042	2.050	2.584				
O	0.605	4.681	-1.883	W4_7	W15_1	W7_1	W4_2_3
H	0.594	5.576	-2.224				
H	-0.212	4.603	-1.391				
O	4.161	2.167	2.715	W4_8	W7_4	W2_6	W4_3_3

H	4.288	3.015	2.291				
H	3.211	2.058	2.756				
O	5.393	0.597	-5.509	W4_9	W5_1	W4_4	W4_4_3
H	4.798	1.280	-5.200				
H	6.111	0.600	-4.876				
O	1.273	5.158	1.849	W4_10	W2_1	W2_5	W4_1_4
H	1.247	6.060	2.167				
H	2.055	5.119	1.299				
O	3.668	4.749	-3.096	W4_11	W15_2	W2_7	W4_2_2
H	4.516	5.175	-3.223				
H	3.036	5.349	-3.492				
O	-1.138	4.410	0.264	W4_12	W2_4	W7_4	W4_1_3
H	-0.296	4.681	0.630				
H	-1.764	4.543	0.976				
O	4.471	4.569	1.735	W4_13	W7_1	W2_1	W4_2_1
H	4.387	4.386	0.800				
H	4.671	5.504	1.779				
O	3.379	2.099	-4.312	W4_14	W15_3	W4_3	W4_4_1
H	3.524	2.978	-3.963				
H	3.114	1.580	-3.553				
O	3.780	0.980	-0.108	W4_15	W7_3	W2_8	W4_4_4
H	3.615	0.633	0.769				
H	3.949	1.912	0.031				
O	3.143	3.244	5.774	W4_16	W16_4	W8_1	W4_3_1
H	3.618	3.046	6.581				
H	2.926	2.388	5.407				

Cluster 3

Element	X	Y	Z	hmbe_4_16	hmbe_16_4	hmbe_8_8	hmbe_4_4_4
O	-4.642	-4.693	-1.329	W1_1	W8_2	W7_2	W1_1_1
H	-4.016	-4.484	-0.636				
H	-5.439	-4.223	-1.083				
O	-3.767	0.219	-3.382	W1_2	W12_4	W7_4	W1_2_3
H	-3.364	0.404	-4.230				

H	-4.650	0.582	-3.451				
O	-6.287	-2.043	3.080	W1_3	W11_1	W4_8	W1_3_4
H	-5.339	-1.937	2.996				
H	-6.628	-1.842	2.209				
O	-4.824	0.936	1.171	W1_4	W2_3	W4_5	W1_4_4
H	-4.728	0.032	1.469				
H	-5.255	1.385	1.899				
O	-5.065	3.913	-5.294	W1_5	W7_4	W6_8	W1_2_2
H	-5.950	3.645	-5.542				
H	-5.103	4.017	-4.343				
O	-6.832	-1.875	0.504	W1_6	W2_2	W7_7	W1_1_2
H	-6.582	-1.534	-0.355				
H	-7.020	-2.800	0.349				
O	-3.817	-1.204	3.065	W1_7	W11_4	W4_2	W1_3_2
H	-3.980	-1.572	3.933				
H	-3.483	-0.323	3.237				
O	-5.140	4.897	-2.561	W1_8	W13_4	W6_7	W1_4_1
H	-5.240	5.334	-1.716				
H	-4.219	4.635	-2.588				
O	-4.353	-2.244	5.653	W1_9	W11_3	W8_1	W1_3_3
H	-3.821	-3.040	5.649				
H	-4.476	-2.042	6.580				
O	-5.678	-0.824	-5.291	W1_10	W5_1	W7_1	W1_2_1
H	-5.931	-0.249	-4.568				
H	-6.231	-0.548	-6.022				
O	-2.895	0.401	-0.754	W1_11	W12_1	W4_3	W1_4_3
H	-3.745	0.427	-0.315				
H	-3.108	0.377	-1.687				
O	-6.213	2.015	3.155	W1_12	W15_1	W4_4	W1_3_1
H	-7.135	2.206	2.984				
H	-5.929	2.711	3.746				
O	-7.198	1.503	-0.411	W1_13	W2_1	W4_1	W1_4_2
H	-6.476	1.533	0.216				
H	-7.857	0.950	0.007				
O	-5.946	-1.692	-1.989	W1_14	W2_4	W7_3	W1_1_3
H	-5.203	-1.138	-2.228				
H	-6.248	-2.059	-2.820				
O	-3.049	-3.998	0.696	W1_15	W8_4	W5_8	W1_1_4
H	-2.229	-3.986	0.203				
H	-3.063	-3.161	1.161				
O	-6.242	0.917	-3.386	W1_16	W7_2	W7_8	W1_2_4

H	-6.665	1.068	-2.541				
H	-6.817	1.342	-4.022				
O	1.299	1.704	-6.283	W2_1	W10_4	W6_5	W2_1_4
H	2.020	1.961	-6.858				
H	1.235	2.416	-5.646				
O	-0.503	-4.279	-5.071	W2_2	W5_3	W2_4	W2_4_1
H	-1.044	-4.698	-5.741				
H	-0.919	-4.519	-4.243				
O	4.661	0.933	-3.946	W2_3	W1_2	W2_5	W2_1_2
H	4.746	0.159	-3.390				
H	4.415	0.587	-4.804				
O	-2.639	0.441	-7.407	W2_4	W7_3	W6_6	W2_3_4
H	-2.747	1.303	-7.004				
H	-2.188	-0.080	-6.742				
O	0.105	-1.133	-3.174	W2_5	W12_3	W2_8	W2_4_2
H	0.064	-0.237	-2.841				
H	-0.304	-1.663	-2.490				
O	2.231	-4.072	-4.541	W2_6	W1_1	W2_3	W2_2_1
H	2.056	-5.013	-4.540				
H	1.408	-3.676	-4.829				
O	-3.525	-3.014	-5.944	W2_7	W5_4	W7_5	W2_4_3
H	-4.313	-2.476	-6.018				
H	-3.662	-3.533	-5.151				
O	1.317	1.774	-2.366	W2_8	W10_3	W1_3	W2_1_3
H	2.139	1.603	-1.906				
H	1.576	2.273	-3.141				
O	4.681	-2.401	-4.765	W2_9	W1_4	W2_1	W2_2_4
H	4.053	-3.075	-4.504				
H	4.324	-2.044	-5.579				
O	-1.272	-1.148	-5.668	W2_10	W5_2	W2_6	W2_3_3
H	-1.943	-1.774	-5.398				
H	-0.444	-1.568	-5.435				
O	3.050	-3.925	-1.696	W2_11	W16_3	W5_6	W2_2_3
H	2.427	-3.909	-2.423				
H	2.519	-3.743	-0.921				
O	-1.949	-4.702	-2.666	W2_12	W8_1	W7_6	W2_4_4
H	-2.814	-4.409	-2.379				
H	-1.335	-4.166	-2.163				
O	1.936	4.117	-4.537	W2_13	W10_1	W6_3	W2_1_1
H	2.569	3.837	-5.198				
H	2.382	4.818	-4.061				

O	-3.167	2.956	-6.899	W2_14	W7_1	W6_1	W2_3_1
H	-3.650	3.164	-6.099				
H	-2.878	3.805	-7.233				
O	0.411	-0.250	-8.080	W2_15	W10_2	W2_7	W2_3_2
H	0.848	0.567	-7.842				
H	-0.296	-0.339	-7.441				
O	1.742	-0.972	-5.315	W2_16	W1_3	W2_2	W2_2_2
H	1.106	-0.378	-5.715				
H	1.466	-1.037	-4.401				
O	0.023	-5.790	1.028	W3_1	W3_4	W5_3	W3_3_4
H	-0.273	-5.039	1.544				
H	0.715	-6.187	1.558				
O	4.587	-0.326	4.011	W3_2	W9_1	W8_7	W3_1_2
H	5.373	-0.866	4.095				
H	4.665	0.322	4.712				
O	-0.376	-3.650	2.985	W3_3	W3_3	W8_5	W3_4_2
H	-0.750	-4.169	3.697				
H	0.527	-3.485	3.257				
O	1.713	-2.327	1.001	W3_4	W16_4	W5_2	W3_4_3
H	1.512	-1.442	0.697				
H	1.820	-2.237	1.948				
O	-2.729	-6.247	2.887	W3_5	W3_1	W5_1	W3_3_1
H	-2.827	-6.815	2.123				
H	-2.843	-5.361	2.544				
O	5.649	-2.489	0.240	W3_6	W16_2	W1_1	W3_1_1
H	5.575	-1.635	0.667				
H	4.977	-2.475	-0.442				
O	0.362	0.183	3.998	W3_7	W14_1	W8_4	W3_4_1
H	0.794	-0.021	4.826				
H	1.061	0.139	3.345				
O	-1.667	-1.714	1.442	W3_8	W11_2	W4_7	W3_4_4
H	-2.413	-1.416	1.963				
H	-1.151	-2.247	2.046				
O	-0.484	-7.098	-1.400	W3_9	W3_2	W5_5	W3_3_2
H	-0.282	-6.686	-0.560				
H	-1.189	-7.714	-1.202				
O	-0.614	-2.959	-0.942	W3_10	W8_3	W5_4	W3_3_3
H	0.096	-3.554	-0.701				
H	-0.629	-2.307	-0.241				
O	5.153	0.301	1.331	W3_11	W9_4	W1_7	W3_1_4
H	4.465	0.848	0.951				

H	4.850	0.117	2.220				
O	-0.460	-3.201	6.344	W3_12	W4_4	W8_6	W3_2_4
H	-0.221	-2.293	6.157				
H	0.237	-3.720	5.942				
O	2.994	-4.684	1.575	W3_13	W16_1	W5_7	W3_1_3
H	2.775	-3.791	1.308				
H	3.945	-4.673	1.687				
O	1.405	-0.625	6.309	W3_14	W4_1	W8_2	W3_2_1
H	1.418	-0.314	7.214				
H	2.113	-1.267	6.265				
O	2.601	-2.265	3.826	W3_15	W4_3	W8_3	W3_2_3
H	3.154	-2.954	4.193				
H	3.202	-1.539	3.658				
O	1.287	-4.965	4.895	W3_16	W4_2	W8_8	W3_2_2
H	1.202	-5.875	4.611				
H	2.195	-4.736	4.698				
O	-1.079	3.667	3.455	W4_1	W15_4	W3_3	W4_3_4
H	-1.341	3.271	2.624				
H	-1.885	4.048	3.803				
O	-0.157	3.365	-0.878	W4_2	W6_2	W1_8	W4_1_3
H	0.388	2.703	-1.303				
H	0.467	3.999	-0.524				
O	1.983	0.919	1.803	W4_3	W14_3	W1_2	W4_1_4
H	1.147	0.860	1.340				
H	2.465	1.605	1.341				
O	-0.911	6.660	1.127	W4_4	W6_1	W3_5	W4_2_3
H	-0.148	6.702	1.703				
H	-0.566	6.334	0.296				
O	-4.269	5.926	-0.165	W4_5	W13_1	W3_4	W4_2_1
H	-4.781	5.353	0.406				
H	-3.487	5.417	-0.378				
O	-1.108	5.304	-4.409	W4_6	W13_2	W6_4	W4_2_2
H	-0.291	4.961	-4.046				
H	-0.840	5.783	-5.193				
O	3.368	3.078	0.384	W4_7	W9_3	W1_5	W4_4_2
H	4.076	3.717	0.299				
H	2.594	3.603	0.588				
O	-3.514	4.412	4.614	W4_8	W15_3	W3_7	W4_3_2
H	-4.469	4.462	4.651				
H	-3.230	4.567	5.515				
O	6.391	2.731	2.235	W4_9	W9_2	W1_4	W4_4_3

H	5.698	3.264	2.623				
H	5.961	1.907	2.006				
O	0.000	0.000	0.000	W4_10	W12_2	W1_6	W4_1_2
H	-0.430	0.564	-0.643				
H	-0.709	-0.297	0.571				
O	-2.047	2.505	1.160	W4_11	W14_2	W3_2	W4_1_1
H	-2.630	1.761	1.012				
H	-1.376	2.423	0.482				
O	-2.360	4.942	-1.743	W4_12	W13_3	W6_2	W4_2_4
H	-2.102	5.294	-2.595				
H	-1.667	4.320	-1.520				
O	1.175	5.830	2.963	W4_13	W6_3	W3_1	W4_4_1
H	1.678	5.682	3.764				
H	0.274	5.611	3.201				
O	1.146	3.073	4.772	W4_14	W14_4	W3_6	W4_3_1
H	1.275	2.153	4.539				
H	0.381	3.343	4.264				
O	-2.212	0.886	3.803	W4_15	W15_2	W4_6	W4_3_3
H	-1.938	1.803	3.820				
H	-1.393	0.391	3.794				
O	1.472	5.205	0.163	W4_16	W6_4	W3_8	W4_4_4
H	1.595	5.328	1.105				
H	1.538	6.086	-0.204				

Cluster 4

Element	X	Y	Z	hmbe_4_16	hmbe_16_4	hmbe_8_8	hmbe_4_4_4
O	5.408	-0.973	-1.110	W1_1	W15_1	W5_3	W1_1_3
H	4.616	-0.454	-0.966				
H	5.831	-0.561	-1.864				
O	2.383	2.643	2.988	W1_2	W5_3	W1_8	W1_3_2
H	2.079	1.759	2.785				
H	2.604	2.613	3.919				
O	2.271	5.303	2.209	W1_3	W5_4	W8_3	W1_4_4
H	2.275	4.388	2.491				
H	2.270	5.805	3.024				
O	-0.177	0.204	2.736	W1_4	W3_3	W1_3	W1_2_4

H	-0.419	0.387	3.644				
H	0.775	0.303	2.716				
O	0.878	3.773	6.602	W1_5	W6_3	W1_5	W1_3_3
H	0.334	4.018	7.350				
H	0.617	4.377	5.907				
O	5.236	-0.391	2.858	W1_6	W2_4	W5_8	W1_1_1
H	6.021	-0.426	3.404				
H	5.555	-0.546	1.969				
O	3.116	2.695	5.738	W1_7	W6_2	W1_2	W1_3_1
H	2.407	3.163	6.178				
H	3.916	3.060	6.117				
O	0.364	3.976	0.179	W1_8	W10_3	W8_4	W1_4_3
H	0.645	3.613	-0.661				
H	1.128	4.457	0.499				
O	5.831	2.073	0.144	W1_9	W12_1	W5_1	W1_1_2
H	6.566	1.736	-0.369				
H	5.651	2.935	-0.233				
O	3.202	6.019	-0.375	W1_10	W5_2	W8_5	W1_4_2
H	4.110	5.732	-0.283				
H	2.826	5.909	0.498				
O	3.169	0.072	-0.279	W1_11	W12_4	W5_7	W1_2_1
H	2.278	0.272	0.009				
H	3.668	0.865	-0.082				
O	5.433	3.395	2.491	W1_12	W5_1	W5_5	W1_4_1
H	5.704	2.823	1.773				
H	4.790	2.880	2.978				
O	0.000	0.000	0.000	W1_13	W12_2	W4_4	W1_2_2
H	-0.376	0.356	0.805				
H	0.259	-0.892	0.230				
O	3.287	-0.071	6.259	W1_14	W6_4	W1_7	W1_3_4
H	2.850	0.644	5.796				
H	4.140	0.287	6.504				
O	6.052	-3.086	1.705	W1_15	W2_1	W5_2	W1_1_4
H	5.761	-3.116	2.616				
H	5.284	-2.789	1.217				
O	2.496	-0.001	3.021	W1_16	W2_2	W1_4	W1_2_3
H	2.256	-0.880	2.729				
H	3.446	-0.035	3.132				
O	-0.684	5.037	-2.894	W2_1	W10_2	W8_2	W2_4_2
H	-1.387	4.549	-2.465				
H	-0.758	5.924	-2.542				

O	0.299	-4.209	-3.454	W2_2	W8_4	W3_2	W2_2_4
H	0.193	-4.283	-2.506				
H	0.390	-3.269	-3.613				
O	-4.384	-1.234	-5.699	W2_3	W1_4	W2_5	W2_1_1
H	-4.298	-0.419	-6.193				
H	-3.491	-1.442	-5.421				
O	0.281	2.520	-3.979	W2_4	W4_3	W8_6	W2_4_3
H	1.023	2.636	-4.572				
H	-0.113	3.390	-3.916				
O	4.373	0.599	-5.666	W2_5	W12_3	W5_4	W2_3_2
H	5.008	-0.118	-5.665				
H	4.852	1.344	-5.304				
O	-6.307	0.956	-4.131	W2_6	W1_1	W2_1	W2_1_4
H	-5.609	1.082	-3.488				
H	-6.262	0.026	-4.355				
O	-0.961	-0.285	-7.686	W2_7	W1_3	W2_4	W2_3_1
H	-0.240	0.011	-7.131				
H	-1.419	-0.938	-7.157				
O	1.027	6.698	-4.025	W2_8	W10_4	W8_7	W2_4_4
H	0.689	6.870	-4.904				
H	0.504	5.963	-3.706				
O	-2.785	1.929	-4.872	W2_9	W4_1	W2_3	W2_1_2
H	-2.527	2.748	-5.296				
H	-1.959	1.531	-4.598				
O	-0.871	0.428	-2.545	W2_10	W4_4	W2_7	W2_1_3
H	-0.427	1.166	-2.964				
H	-0.734	0.567	-1.608				
O	0.387	-1.723	-3.965	W2_11	W8_2	W2_2	W2_3_3
H	0.027	-1.139	-3.298				
H	1.143	-1.252	-4.314				
O	1.626	0.040	-6.009	W2_12	W4_2	W2_6	W2_3_4
H	2.492	0.153	-5.616				
H	1.790	0.027	-6.952				
O	3.487	5.325	-3.264	W2_13	W10_1	W8_1	W2_4_1
H	3.246	5.447	-2.346				
H	2.928	5.938	-3.742				
O	-1.991	-4.778	-4.833	W2_14	W8_1	W3_6	W2_2_1
H	-1.144	-4.677	-4.398				
H	-2.132	-5.724	-4.869				
O	1.990	-4.957	-5.284	W2_15	W8_3	W3_8	W2_2_2
H	1.481	-4.670	-6.042				

H	1.540	-4.570	-4.533				
O	-1.899	-1.966	-5.341	W2_16	W1_2	W2_8	W2_2_3
H	-1.844	-2.908	-5.181				
H	-1.310	-1.578	-4.695				
O	-1.311	1.385	5.190	W3_1	W9_3	W1_6	W3_2_2
H	-0.502	1.580	5.662				
H	-1.984	1.355	5.869				
O	-6.192	3.183	-0.703	W3_2	W14_2	W7_1	W3_3_2
H	-5.451	3.086	-1.302				
H	-5.791	3.377	0.145				
O	-5.346	1.562	2.723	W3_3	W7_2	W7_3	W3_4_2
H	-5.614	0.645	2.794				
H	-4.399	1.524	2.585				
O	-3.474	-0.183	-1.495	W3_4	W16_3	W4_6	W3_1_3
H	-3.719	0.683	-1.819				
H	-2.622	-0.357	-1.896				
O	-3.919	5.608	-0.093	W3_5	W14_1	W7_4	W3_3_1
H	-4.389	4.969	0.443				
H	-3.870	6.392	0.455				
O	-4.025	3.605	5.351	W3_6	W7_1	W7_2	W3_4_1
H	-3.931	3.225	4.477				
H	-4.589	2.990	5.820				
O	-3.848	-0.889	3.910	W3_7	W3_2	W4_7	W3_2_1
H	-4.713	-0.555	4.148				
H	-4.023	-1.733	3.495				
O	-7.732	2.656	3.356	W3_8	W7_3	W7_5	W3_4_3
H	-6.910	2.260	3.065				
H	-8.062	2.060	4.028				
O	-6.909	-1.557	0.035	W3_9	W16_2	W4_5	W3_1_2
H	-7.461	-0.936	0.510				
H	-6.962	-2.365	0.546				
O	-5.650	-1.618	-2.479	W3_10	W16_1	W4_3	W3_1_1
H	-4.940	-1.107	-2.091				
H	-6.183	-1.893	-1.733				
O	-2.789	2.099	2.754	W3_11	W3_1	W7_7	W3_2_3
H	-2.551	1.743	3.610				
H	-2.045	1.887	2.190				
O	-3.982	2.583	-2.263	W3_12	W14_3	W7_6	W3_3_3
H	-3.300	2.736	-1.610				
H	-3.681	3.054	-3.040				
O	-3.694	-1.478	6.683	W3_13	W9_1	W4_1	W3_2_4

H	-3.475	-0.857	5.988				
H	-4.522	-1.157	7.039				
O	-5.032	3.886	1.704	W3_14	W7_4	W7_8	W3_4_4
H	-4.928	4.619	2.311				
H	-5.273	3.146	2.261				
O	-4.303	-1.381	1.004	W3_15	W16_4	W4_8	W3_1_4
H	-5.242	-1.452	0.829				
H	-3.957	-0.889	0.259				
O	-2.174	3.879	-0.808	W3_16	W14_4	W8_8	W3_3_4
H	-2.849	4.523	-0.594				
H	-1.439	4.102	-0.236				
O	-0.092	-7.145	-0.330	W4_1	W11_4	W3_7	W4_2_2
H	0.701	-7.185	-0.865				
H	-0.387	-6.237	-0.407				
O	2.167	-5.459	4.829	W4_2	W13_3	W6_1	W4_2_1
H	2.747	-6.007	5.358				
H	2.268	-5.792	3.938				
O	-1.913	-1.761	1.737	W4_3	W3_4	W4_2	W4_1_2
H	-2.854	-1.719	1.563				
H	-1.727	-0.968	2.241				
O	0.719	-1.023	7.549	W4_4	W6_1	W1_1	W4_3_1
H	1.657	-0.844	7.622				
H	0.662	-1.744	6.921				
O	-1.295	-5.074	4.314	W4_5	W13_2	W6_6	W4_3_2
H	-1.561	-4.724	5.164				
H	-1.012	-5.969	4.501				
O	3.450	-5.436	-2.155	W4_6	W15_2	W3_3	W4_4_4
H	3.919	-4.872	-2.771				
H	4.130	-5.768	-1.569				
O	2.443	-2.553	2.062	W4_7	W15_3	W6_3	W4_4_3
H	1.665	-3.107	1.997				
H	2.885	-2.661	1.219				
O	-0.823	-8.168	2.528	W4_8	W11_2	W3_4	W4_2_3
H	-0.603	-7.856	1.651				
H	-1.768	-8.031	2.602				
O	2.346	-6.582	2.292	W4_9	W13_1	W6_7	W4_2_4
H	1.402	-6.586	2.133				
H	2.667	-7.368	1.849				
O	-2.033	-3.805	6.690	W4_10	W9_4	W6_5	W4_3_3
H	-2.278	-4.151	7.548				
H	-2.761	-3.235	6.444				

O	-0.240	-4.021	2.138	W4_11	W13_4	W6_4	W4_1_4
H	-0.809	-3.271	1.963				
H	-0.531	-4.349	2.989				
O	-2.614	-6.956	-1.858	W4_12	W11_1	W3_1	W4_1_3
H	-2.086	-7.120	-2.640				
H	-2.305	-7.600	-1.221				
O	0.802	-3.449	6.148	W4_13	W9_2	W6_2	W4_3_4
H	-0.148	-3.562	6.175				
H	1.088	-3.991	5.413				
O	4.147	-3.272	4.000	W4_14	W2_3	W6_8	W4_4_1
H	3.518	-2.749	3.504				
H	3.608	-3.813	4.577				
O	-0.739	-4.501	-0.569	W4_15	W11_3	W3_5	W4_1_1
H	-0.427	-4.321	0.318				
H	-1.614	-4.113	-0.599				
O	3.636	-3.467	-0.125	W4_16	W15_4	W5_6	W4_4_2
H	3.106	-4.112	-0.593				
H	3.898	-2.838	-0.797				

Cluster 5

Element	X	Y	Z	hmbe_4_16	hmbe_16_4	hmbe_8_8	hmbe_4_4_4
O	-1.417	-3.013	-5.621	W1_1	W8_1	W4_3	W1_1_1
H	-0.471	-3.060	-5.483				
H	-1.526	-3.095	-6.568				
O	-4.757	-4.893	1.271	W1_2	W4_1	W1_4	W1_2_4
H	-5.068	-4.263	0.621				
H	-3.979	-5.284	0.874				
O	0.397	-3.344	-1.107	W1_3	W10_2	W4_5	W1_3_2
H	0.562	-3.082	-0.202				
H	1.137	-3.907	-1.333				
O	3.681	-5.942	-1.954	W1_4	W6_1	W4_4	W1_4_2
H	3.877	-6.256	-2.837				
H	3.684	-4.988	-2.033				
O	0.731	-6.823	0.419	W1_5	W9_1	W4_6	W1_3_4
H	0.190	-7.610	0.482				

H	0.302	-6.189	0.993				
O	-6.609	-2.018	-0.932	W1_6	W11_1	W1_7	W1_2_3
H	-5.735	-1.685	-0.728				
H	-6.935	-1.431	-1.614				
O	-3.196	-2.787	-3.636	W1_7	W8_4	W1_6	W1_1_4
H	-2.735	-3.124	-4.405				
H	-2.905	-1.878	-3.562				
O	0.509	-6.301	-5.150	W1_8	W6_3	W4_7	W1_4_3
H	0.803	-5.391	-5.113				
H	0.559	-6.607	-4.245				
O	-5.397	-4.528	-1.685	W1_9	W4_4	W1_2	W1_2_1
H	-4.467	-4.364	-1.841				
H	-5.803	-3.662	-1.721				
O	-3.137	-3.322	3.818	W1_10	W2_3	W5_3	W1_2_2
H	-3.208	-3.722	2.951				
H	-4.010	-3.419	4.199				
O	-2.781	-2.634	-0.449	W1_11	W4_3	W1_3	W1_3_3
H	-2.299	-2.338	-1.221				
H	-2.664	-3.585	-0.444				
O	-3.392	-5.932	-4.011	W1_12	W4_2	W1_5	W1_1_2
H	-3.863	-5.144	-3.737				
H	-2.680	-5.606	-4.561				
O	0.010	-6.902	-2.416	W1_13	W9_4	W4_1	W1_4_1
H	0.268	-6.741	-1.508				
H	-0.947	-6.912	-2.395				
O	2.638	-3.864	-3.733	W1_14	W6_4	W4_8	W1_4_4
H	2.388	-3.374	-4.517				
H	3.182	-4.580	-4.061				
O	-1.043	-1.287	-2.370	W1_15	W10_3	W7_4	W1_1_3
H	-0.629	-2.089	-2.052				
H	-0.971	-1.341	-3.323				
O	-2.659	-5.773	0.075	W1_16	W9_3	W1_8	W1_3_1
H	-2.336	-6.438	0.684				
H	-2.865	-6.259	-0.723				
O	-0.283	0.284	-5.252	W2_1	W1_2	W7_6	W2_4_3
H	0.456	-0.289	-5.050				
H	0.105	1.155	-5.337				
O	-7.454	2.640	-0.540	W2_2	W11_2	W3_1	W2_2_3
H	-8.002	2.549	-1.320				
H	-6.557	2.632	-0.875				
O	0.000	0.000	0.000	W2_3	W10_4	W6_7	W2_3_4

H	-0.341	-0.689	-0.571				
H	-0.759	0.288	0.507				
O	-5.594	-0.924	-5.157	W2_4	W8_2	W1_1	W2_4_1
H	-5.288	-0.070	-5.462				
H	-4.926	-1.210	-4.533				
O	2.106	1.730	-1.051	W2_5	W3_3	W2_5	W2_3_3
H	2.174	2.505	-0.493				
H	1.305	1.295	-0.760				
O	0.467	3.081	-4.668	W2_6	W1_1	W7_8	W2_1_1
H	-0.324	3.344	-5.139				
H	0.304	3.338	-3.761				
O	-4.671	2.642	-1.517	W2_7	W11_4	W3_6	W2_2_4
H	-4.173	3.162	-0.886				
H	-4.079	1.933	-1.765				
O	-2.427	0.826	-1.664	W2_8	W10_1	W3_5	W2_3_1
H	-1.737	1.375	-2.037				
H	-2.146	-0.070	-1.846				
O	2.412	-0.822	-4.515	W2_9	W6_2	W7_7	W2_1_2
H	3.060	-0.506	-3.885				
H	2.932	-1.179	-5.235				
O	-4.432	-0.761	0.071	W2_10	W5_3	W6_6	W2_2_2
H	-3.853	-1.509	0.217				
H	-3.843	-0.041	-0.152				
O	-0.468	3.790	-2.012	W2_11	W14_2	W3_2	W2_3_2
H	-0.553	3.570	-1.084				
H	-0.321	4.736	-2.022				
O	-2.910	-0.324	-5.607	W2_12	W8_3	W7_1	W2_4_4
H	-2.015	-0.008	-5.477				
H	-3.025	-0.336	-6.557				
O	-1.866	3.479	-5.737	W2_13	W14_3	W3_4	W2_4_2
H	-2.475	4.215	-5.805				
H	-2.069	2.928	-6.493				
O	2.495	4.169	-2.839	W2_14	W1_3	W7_2	W2_1_3
H	2.121	3.907	-3.680				
H	2.050	3.616	-2.197				
O	-5.268	4.361	-3.444	W2_15	W14_1	W3_8	W2_2_1
H	-4.968	3.616	-2.923				
H	-5.646	4.964	-2.803				
O	2.645	2.860	-5.820	W2_16	W1_4	W7_5	W2_1_4
H	1.894	2.658	-5.262				
H	2.262	3.240	-6.610				

O	-6.508	0.235	2.072	W3_1	W11_3	W6_5	W3_4_1
H	-6.801	1.133	1.915				
H	-6.016	0.002	1.285				
O	-0.209	4.711	0.895	W3_2	W12_2	W8_3	W3_3_3
H	0.337	5.476	1.075				
H	0.384	3.965	0.991				
O	-3.644	0.825	5.114	W3_3	W16_1	W6_4	W3_4_3
H	-3.803	1.268	5.947				
H	-3.128	0.053	5.347				
O	-2.291	6.737	2.515	W3_4	W12_1	W8_7	W3_3_2
H	-1.424	7.078	2.298				
H	-2.122	6.034	3.143				
O	1.874	4.976	4.676	W3_5	W7_1	W8_8	W3_2_1
H	2.007	5.920	4.764				
H	1.013	4.892	4.269				
O	-3.748	6.510	-1.333	W3_6	W14_4	W3_3	W3_3_1
H	-3.747	5.675	-0.865				
H	-4.666	6.654	-1.563				
O	-0.863	2.606	3.857	W3_7	W16_3	W8_6	W3_1_2
H	-1.527	3.294	3.809				
H	-0.195	2.872	3.225				
O	2.226	6.545	0.733	W3_8	W7_2	W8_5	W3_2_2
H	2.155	7.495	0.824				
H	2.745	6.417	-0.061				
O	-3.840	3.859	5.231	W3_9	W5_4	W6_8	W3_1_3
H	-4.572	3.774	5.843				
H	-4.230	4.239	4.444				
O	-3.760	4.346	0.472	W3_10	W12_3	W3_7	W3_3_4
H	-3.043	4.788	0.927				
H	-4.250	3.906	1.166				
O	-0.788	2.289	6.830	W3_11	W16_2	W6_1	W3_1_4
H	-0.022	1.839	6.474				
H	-1.349	2.448	6.071				
O	-4.226	2.439	2.613	W3_12	W5_1	W6_2	W3_4_4
H	-3.791	2.041	3.367				
H	-5.151	2.223	2.728				
O	1.244	2.605	1.739	W3_13	W7_3	W8_4	W3_2_3
H	1.389	1.729	2.097				
H	2.019	3.102	2.000				
O	-1.791	0.478	2.196	W3_14	W5_2	W6_3	W3_4_2
H	-1.420	1.187	2.721				

H	-2.737	0.562	2.314				
O	-1.353	5.310	4.789	W3_15	W12_4	W8_1	W3_1_1
H	-1.623	6.159	5.139				
H	-2.009	4.695	5.118				
O	3.265	4.364	2.514	W3_16	W7_4	W8_2	W3_2_4
H	3.159	5.254	2.178				
H	2.915	4.401	3.404				
O	4.528	-1.076	0.451	W4_1	W15_4	W2_2	W4_1_4
H	5.206	-1.398	1.045				
H	4.695	-1.532	-0.373				
O	1.651	-2.175	3.972	W4_2	W13_2	W5_2	W4_4_3
H	0.740	-2.189	4.266				
H	1.600	-2.328	3.028				
O	-1.999	-2.223	5.982	W4_3	W2_1	W5_6	W4_2_1
H	-2.494	-2.644	6.685				
H	-2.464	-2.468	5.182				
O	2.111	0.478	3.223	W4_4	W3_1	W2_3	W4_3_3
H	3.001	0.351	2.893				
H	1.950	-0.284	3.778				
O	5.561	1.973	4.240	W4_5	W3_4	W2_8	W4_3_4
H	5.612	1.147	3.760				
H	5.215	1.731	5.099				
O	4.576	-2.106	-2.015	W4_6	W15_1	W4_2	W4_1_1
H	4.340	-1.225	-2.305				
H	3.990	-2.684	-2.503				
O	2.237	-5.804	4.408	W4_7	W13_4	W5_7	W4_4_4
H	2.836	-5.093	4.637				
H	1.584	-5.395	3.840				
O	-0.369	-5.027	2.605	W4_8	W9_2	W5_8	W4_2_2
H	-0.231	-4.162	2.220				
H	-0.333	-4.878	3.550				
O	4.259	-3.877	3.835	W4_9	W13_1	W5_4	W4_4_1
H	4.729	-3.991	3.009				
H	3.605	-3.204	3.648				
O	1.786	-2.001	0.976	W4_10	W3_2	W2_4	W4_4_2
H	2.713	-1.787	0.868				
H	1.333	-1.165	0.867				
O	-0.787	-2.099	2.391	W4_11	W2_2	W5_5	W4_2_3
H	-0.912	-1.197	2.098				
H	-1.606	-2.324	2.833				
O	4.045	0.287	-2.573	W4_12	W15_3	W7_3	W4_1_3

H	3.434	0.734	-1.987				
H	4.752	0.917	-2.709				
O	-0.234	-4.418	5.222	W4_13	W2_4	W5_1	W4_2_4
H	-0.766	-3.676	5.512				
H	-0.724	-5.190	5.504				
O	5.950	1.186	0.707	W4_14	W15_2	W2_6	W4_1_2
H	6.357	1.297	-0.153				
H	5.199	0.615	0.545				
O	0.988	0.966	5.669	W4_15	W16_4	W2_7	W4_3_2
H	1.585	0.418	6.179				
H	1.349	0.954	4.783				
O	4.755	-0.242	3.224	W4_16	W13_3	W2_1	W4_3_1
H	5.434	-0.693	2.723				
H	4.760	-0.673	4.078				

Cluster 6

Element	X	Y	Z	hmbe_4_16	hmbe_16_4	hmbe_8_8	hmbe_4_4_4
O	3.910	-3.049	-2.189	W1_1	W10_2	W8_2	W1_1_4
H	4.779	-3.094	-2.589				
H	3.420	-2.445	-2.747				
O	-3.713	-0.632	-1.641	W1_2	W16_1	W4_8	W1_4_4
H	-3.964	0.278	-1.798				
H	-2.835	-0.707	-2.015				
O	0.228	-6.940	-0.822	W1_3	W8_1	W8_5	W1_3_1
H	1.027	-7.050	-1.337				
H	-0.208	-6.183	-1.214				
O	0.285	-0.362	-6.617	W1_4	W2_3	W4_1	W1_1_3
H	1.224	-0.477	-6.764				
H	-0.015	-1.214	-6.300				
O	-3.580	-6.694	1.054	W1_5	W13_1	W7_1	W1_2_2
H	-3.393	-6.673	0.115				
H	-3.474	-7.613	1.299				
O	-0.674	-5.342	-3.021	W1_6	W8_3	W8_6	W1_2_3
H	0.013	-5.591	-3.639				
H	-0.889	-4.438	-3.253				

O	-4.594	-3.126	-0.151	W1_7	W5_4	W7_3	W1_2_4
H	-4.028	-2.682	-0.782				
H	-4.505	-4.056	-0.361				
O	1.543	-2.270	-3.516	W1_8	W2_4	W8_3	W1_1_2
H	1.878	-1.373	-3.513				
H	1.059	-2.348	-2.693				
O	-2.673	-1.218	-5.423	W1_9	W2_1	W4_4	W1_4_3
H	-3.030	-0.448	-5.867				
H	-2.217	-1.701	-6.112				
O	-0.225	-2.732	-1.406	W1_10	W8_2	W2_6	W1_3_2
H	-0.302	-3.668	-1.591				
H	-0.211	-2.677	-0.450				
O	0.835	-4.534	1.226	W1_11	W14_2	W2_5	W1_3_3
H	0.235	-3.788	1.225				
H	0.284	-5.285	1.445				
O	-3.646	-4.764	-2.369	W1_12	W5_2	W7_4	W1_2_1
H	-3.037	-5.501	-2.325				
H	-3.962	-4.768	-3.272				
O	1.555	-3.554	-5.788	W1_13	W2_2	W8_7	W1_1_1
H	1.432	-4.491	-5.633				
H	1.686	-3.181	-4.916				
O	-4.738	-2.139	-3.922	W1_14	W16_3	W4_7	W1_4_1
H	-4.039	-2.088	-4.574				
H	-4.368	-1.729	-3.141				
O	2.441	-6.525	-2.385	W1_15	W8_4	W8_1	W1_3_4
H	2.116	-6.388	-3.275				
H	2.739	-5.660	-2.103				
O	-1.337	-0.721	-3.060	W1_16	W16_4	W4_2	W1_4_2
H	-1.796	-1.127	-3.795				
H	-1.076	-1.456	-2.505				
O	5.042	0.801	1.378	W2_1	W12_3	W1_5	W2_1_3
H	5.996	0.795	1.456				
H	4.845	0.068	0.793				
O	0.074	-1.558	3.540	W2_2	W1_3	W2_4	W2_2_1
H	1.016	-1.676	3.667				
H	-0.089	-0.649	3.792				
O	3.004	-4.437	2.852	W2_3	W1_4	W2_1	W2_4_4
H	3.750	-4.232	2.288				
H	2.242	-4.373	2.276				
O	1.740	1.976	2.313	W2_4	W6_3	W3_5	W2_3_3
H	2.155	1.768	1.476				

H	1.001	1.371	2.372				
O	5.601	2.988	-1.392	W2_5	W10_1	W1_1	W2_1_4
H	5.728	3.445	-2.223				
H	5.748	2.065	-1.601				
O	3.306	3.853	0.280	W2_6	W11_2	W1_7	W2_1_1
H	3.456	4.703	-0.135				
H	3.961	3.276	-0.113				
O	2.574	0.302	-3.008	W2_7	W10_3	W1_3	W2_1_2
H	1.863	0.938	-2.924				
H	3.367	0.836	-3.066				
O	0.000	0.000	0.000	W2_8	W14_4	W2_8	W2_2_2
H	-0.614	0.342	0.650				
H	0.154	-0.906	0.269				
O	5.584	-3.816	1.514	W2_9	W12_4	W8_4	W2_4_1
H	5.631	-3.109	0.872				
H	5.924	-4.583	1.053				
O	2.281	-1.296	1.634	W2_10	W12_2	W2_3	W2_2_3
H	2.935	-1.182	2.324				
H	2.713	-1.847	0.982				
O	3.624	4.312	3.310	W2_11	W6_2	W3_2	W2_3_2
H	3.696	3.983	2.414				
H	2.918	4.957	3.273				
O	2.089	1.893	5.255	W2_12	W6_1	W3_4	W2_3_1
H	2.457	2.487	4.600				
H	2.578	1.078	5.141				
O	2.717	-1.169	4.549	W2_13	W1_1	W3_3	W2_2_4
H	2.857	-1.615	5.384				
H	3.559	-0.759	4.352				
O	5.169	-1.731	-0.137	W2_14	W12_1	W8_8	W2_4_3
H	4.690	-2.264	-0.771				
H	5.852	-1.300	-0.653				
O	2.529	-5.222	5.126	W2_15	W1_2	W2_7	W2_4_2
H	2.717	-4.972	4.221				
H	3.283	-4.906	5.624				
O	4.526	0.969	3.891	W2_16	W6_4	W3_6	W2_3_4
H	4.386	0.815	2.957				
H	5.309	1.518	3.929				
O	-1.988	4.993	2.909	W3_1	W3_3	W6_3	W3_1_3
H	-1.086	4.673	2.881				
H	-2.405	4.600	2.142				
O	-2.460	2.699	-3.022	W3_2	W15_2	W4_3	W3_2_4

H	-1.673	2.248	-2.714				
H	-2.327	2.794	-3.965				
O	0.761	4.126	-1.795	W3_3	W11_4	W1_2	W3_3_2
H	0.681	3.196	-2.009				
H	1.336	4.148	-1.030				
O	-3.411	2.614	0.833	W3_4	W7_3	W6_5	W3_4_3
H	-3.049	1.728	0.836				
H	-4.130	2.577	0.202				
O	-2.155	7.808	1.957	W3_5	W3_2	W6_7	W3_1_2
H	-1.435	8.077	2.528				
H	-2.254	6.870	2.123				
O	3.518	3.309	-4.085	W3_6	W10_4	W1_8	W3_3_4
H	2.779	3.914	-4.155				
H	4.245	3.853	-3.782				
O	-5.671	5.565	0.809	W3_7	W7_2	W6_1	W3_4_2
H	-4.806	5.702	0.423				
H	-5.922	4.684	0.530				
O	1.921	6.549	3.697	W3_8	W3_1	W3_7	W3_1_1
H	1.327	5.880	4.038				
H	1.387	7.342	3.650				
O	-6.389	3.035	-0.050	W3_9	W7_4	W6_4	W3_4_1
H	-5.800	2.439	-0.514				
H	-7.207	2.997	-0.545				
O	0.102	1.351	-2.330	W3_10	W16_2	W4_6	W3_2_2
H	0.273	1.006	-1.453				
H	-0.353	0.641	-2.783				
O	2.124	5.853	-3.536	W3_11	W11_3	W1_6	W3_3_1
H	2.567	6.571	-3.085				
H	1.670	5.377	-2.840				
O	-1.015	6.254	-1.828	W3_12	W11_1	W6_2	W3_3_3
H	-0.684	5.382	-2.041				
H	-0.399	6.592	-1.177				
O	-1.576	3.508	-5.452	W3_13	W15_3	W4_5	W3_2_3
H	-0.989	4.229	-5.225				
H	-2.152	3.870	-6.125				
O	-3.542	5.478	-0.819	W3_14	W15_1	W6_8	W3_4_4
H	-2.774	5.913	-1.189				
H	-3.227	4.611	-0.564				
O	0.447	4.420	2.309	W3_15	W3_4	W1_4	W3_1_4
H	0.947	3.604	2.297				
H	0.712	4.876	1.510				

O	-4.615	2.129	-2.098	W3_16	W15_4	W6_6	W3_2_1
H	-3.711	2.374	-2.297				
H	-5.114	2.409	-2.865				
O	-6.355	0.649	1.489	W4_1	W7_1	W5_2	W4_3_4
H	-6.391	-0.129	0.932				
H	-6.294	1.381	0.875				
O	-0.437	1.203	4.259	W4_2	W9_3	W3_8	W4_1_3
H	0.452	1.556	4.240				
H	-0.820	1.558	5.061				
O	-1.155	-6.184	2.525	W4_3	W13_3	W7_6	W4_2_3
H	-1.648	-6.465	3.296				
H	-1.810	-6.130	1.829				
O	-3.318	0.455	6.939	W4_4	W4_1	W5_1	W4_4_2
H	-4.188	0.798	6.735				
H	-3.208	-0.287	6.344				
O	-3.610	-3.852	3.711	W4_5	W13_4	W7_8	W4_2_1
H	-4.179	-4.430	3.204				
H	-3.176	-4.431	4.337				
O	-1.768	0.350	1.930	W4_6	W14_3	W5_4	W4_1_4
H	-1.449	0.445	2.828				
H	-2.427	-0.342	1.982				
O	-3.603	2.749	4.007	W4_7	W9_4	W5_3	W4_4_4
H	-3.066	3.531	4.133				
H	-3.139	2.244	3.339				
O	-6.840	-2.005	0.128	W4_8	W5_1	W7_7	W4_3_1
H	-7.151	-2.910	0.160				
H	-5.911	-2.078	-0.093				
O	-0.221	0.063	8.095	W4_9	W9_2	W3_1	W4_1_2
H	-0.602	-0.737	7.733				
H	0.211	0.481	7.350				
O	-2.967	-1.550	5.281	W4_10	W4_3	W5_5	W4_1_1
H	-2.136	-1.579	4.806				
H	-3.442	-2.324	4.979				
O	-3.330	-1.856	2.070	W4_11	W5_3	W7_2	W4_3_3
H	-3.363	-2.586	2.689				
H	-3.860	-2.145	1.328				
O	-1.849	2.602	6.187	W4_12	W9_1	W5_6	W4_4_3
H	-2.073	1.953	6.853				
H	-2.657	3.096	6.050				
O	-6.875	0.181	3.854	W4_13	W4_2	W5_7	W4_3_2
H	-6.500	-0.689	3.995				

H	-6.617	0.411	2.961				
O	-5.278	1.831	5.634	W4_14	W4_4	W5_8	W4_4_1
H	-5.854	1.427	4.985				
H	-4.482	2.047	5.148				
O	-0.670	-2.388	1.425	W4_15	W14_1	W2_2	W4_2_2
H	-0.330	-2.123	2.280				
H	-1.380	-1.771	1.247				
O	-5.273	-5.150	2.583	W4_16	W13_2	W7_5	W4_2_4
H	-4.599	-5.561	2.042				
H	-5.830	-4.684	1.960				

Cluster 7

Element	X	Y	Z	hmbe_4_16	hmbe_16_4	hmbe_8_8	hmbe_4_4_4
O	-5.005	-2.479	2.807	W1_1	W3_3	W4_3	W1_4_3
H	-5.809	-2.320	2.313				
H	-4.371	-1.863	2.439				
O	-3.437	-2.553	-0.397	W1_2	W9_1	W2_4	W1_1_1
H	-2.801	-2.251	-1.046				
H	-3.591	-1.792	0.162				
O	-2.194	-5.359	-3.936	W1_3	W2_4	W7_8	W1_3_1
H	-1.843	-5.198	-3.060				
H	-1.443	-5.672	-4.441				
O	-7.365	0.717	-2.446	W1_4	W15_1	W2_5	W1_2_1
H	-7.722	0.218	-1.711				
H	-6.438	0.480	-2.469				
O	-6.933	1.065	1.868	W1_5	W15_2	W4_2	W1_2_2
H	-7.461	0.814	2.625				
H	-7.283	1.915	1.601				
O	-3.751	-5.131	2.443	W1_6	W3_4	W4_5	W1_4_1
H	-3.947	-5.845	3.050				
H	-4.221	-4.377	2.800				
O	-2.138	-1.001	3.463	W1_7	W12_2	W4_6	W1_1_2
H	-1.487	-1.656	3.717				
H	-2.495	-0.683	4.292				
O	-1.516	-5.001	-1.280	W1_8	W9_2	W7_4	W1_3_3

H	-2.079	-4.312	-0.928				
H	-0.663	-4.843	-0.876				
O	-2.203	-7.605	-0.586	W1_9	W2_1	W7_1	W1_3_4
H	-1.558	-8.220	-0.936				
H	-1.953	-6.759	-0.958				
O	-4.992	-0.333	-3.021	W1_10	W15_3	W2_2	W1_2_3
H	-4.690	-1.172	-2.671				
H	-4.397	-0.148	-3.747				
O	-1.706	-1.577	-2.199	W1_11	W9_3	W5_8	W1_3_2
H	-1.706	-1.586	-3.156				
H	-0.796	-1.403	-1.960				
O	-5.105	-3.100	5.373	W1_12	W12_3	W4_1	W1_4_2
H	-5.486	-2.344	5.820				
H	-5.235	-2.920	4.442				
O	-3.880	-0.502	1.270	W1_13	W3_2	W4_4	W1_1_3
H	-3.067	-0.452	1.772				
H	-4.117	0.410	1.106				
O	-5.698	-4.255	0.619	W1_14	W3_1	W4_7	W1_4_4
H	-5.306	-3.427	0.343				
H	-4.958	-4.781	0.923				
O	-1.021	-2.626	0.945	W1_15	W9_4	W5_7	W1_1_4
H	-1.968	-2.725	0.847				
H	-0.904	-2.316	1.843				
O	-6.873	-1.375	1.031	W1_16	W15_4	W4_8	W1_2_4
H	-7.713	-1.140	0.637				
H	-6.478	-0.536	1.270				
O	-0.694	1.739	5.634	W2_1	W7_1	W1_5	W2_4_4
H	-1.255	1.012	5.364				
H	-0.184	1.389	6.366				
O	3.940	5.671	0.786	W2_2	W5_1	W6_5	W2_1_1
H	4.014	5.006	1.471				
H	3.515	5.219	0.058				
O	-1.851	5.334	0.646	W2_3	W1_1	W3_5	W2_2_4
H	-2.789	5.298	0.460				
H	-1.454	5.621	-0.176				
O	1.167	2.697	3.587	W2_4	W13_3	W1_2	W2_1_4
H	1.514	1.832	3.366				
H	0.742	2.574	4.435				
O	2.074	2.348	7.019	W2_5	W7_4	W1_7	W2_4_2
H	1.314	2.027	7.503				
H	2.656	1.591	6.955				

O	-1.457	4.955	3.608	W2_6	W13_4	W3_3	W2_2_1
H	-1.919	5.741	3.900				
H	-1.244	5.129	2.691				
O	2.977	0.776	3.553	W2_7	W5_3	W1_3	W2_3_2
H	3.647	1.400	3.831				
H	3.470	0.014	3.250				
O	-2.693	2.133	1.346	W2_8	W11_1	W3_8	W2_2_3
H	-2.301	1.843	0.522				
H	-2.241	2.952	1.551				
O	-4.599	5.206	1.361	W2_9	W1_3	W3_1	W2_2_2
H	-4.392	4.279	1.479				
H	-4.542	5.578	2.241				
O	4.587	2.691	5.016	W2_10	W5_2	W1_6	W2_3_4
H	4.301	2.469	5.902				
H	5.509	2.435	4.991				
O	4.708	1.057	1.240	W2_11	W10_3	W6_6	W2_3_3
H	4.893	1.846	0.731				
H	5.380	0.432	0.965				
O	1.350	5.922	3.825	W2_12	W13_2	W3_7	W2_1_3
H	1.567	6.067	4.746				
H	0.518	5.449	3.846				
O	3.690	4.043	3.059	W2_13	W5_4	W6_4	W2_3_1
H	4.069	3.905	3.927				
H	2.847	3.591	3.090				
O	-2.503	-0.197	5.897	W2_14	W12_4	W1_8	W2_4_3
H	-2.230	-1.055	6.222				
H	-3.312	-0.007	6.372				
O	-2.986	3.013	5.073	W2_15	W13_1	W3_4	W2_4_1
H	-2.244	2.590	5.505				
H	-2.596	3.491	4.341				
O	0.655	3.167	1.114	W2_16	W11_3	W6_2	W2_1_2
H	1.549	3.281	0.793				
H	0.738	3.176	2.067				
O	3.816	-4.687	-2.310	W3_1	W16_4	W7_5	W3_2_4
H	3.210	-4.120	-2.788				
H	3.553	-4.601	-1.394				
O	2.998	-2.135	3.272	W3_2	W10_4	W8_7	W3_1_4
H	3.764	-1.959	3.818				
H	2.904	-3.088	3.291				
O	0.825	-4.457	0.419	W3_3	W16_3	W7_3	W3_3_3
H	0.805	-4.999	1.208				

H	0.640	-3.571	0.732				
O	-0.351	-3.290	7.055	W3_4	W12_1	W1_1	W3_4_2
H	0.046	-2.437	6.882				
H	-1.271	-3.180	6.814				
O	0.852	-6.345	-3.958	W3_5	W2_2	W7_7	W3_2_3
H	1.198	-7.183	-4.266				
H	0.715	-6.473	-3.019				
O	3.388	-4.984	3.475	W3_6	W14_4	W8_3	W3_3_1
H	4.053	-5.666	3.563				
H	3.181	-4.733	4.376				
O	-0.399	-6.030	2.636	W3_7	W14_3	W7_6	W3_3_2
H	-0.593	-5.995	3.573				
H	-1.114	-6.543	2.262				
O	3.969	-4.059	0.384	W3_8	W16_1	W8_6	W3_2_2
H	3.347	-4.665	0.786				
H	4.806	-4.262	0.801				
O	1.163	-7.028	-0.985	W3_9	W2_3	W7_2	W3_2_1
H	1.098	-6.523	-0.175				
H	1.813	-7.705	-0.794				
O	1.766	-1.041	5.710	W3_10	W7_3	W1_4	W3_4_4
H	2.051	-0.624	4.898				
H	2.208	-0.549	6.401				
O	6.228	-4.012	2.041	W3_11	W10_2	W8_1	W3_1_3
H	6.470	-3.159	1.680				
H	6.767	-4.102	2.826				
O	0.058	-3.008	3.508	W3_12	W7_2	W8_4	W3_4_3
H	0.182	-3.774	4.068				
H	0.936	-2.641	3.405				
O	5.489	-1.460	4.343	W3_13	W10_1	W8_5	W3_1_1
H	6.239	-1.890	3.933				
H	5.640	-1.558	5.283				
O	2.678	-4.075	5.898	W3_14	W14_1	W8_2	W3_4_1
H	3.150	-4.075	6.731				
H	2.293	-3.200	5.841				
O	2.466	-1.474	0.733	W3_15	W16_2	W5_6	W3_1_2
H	2.568	-1.444	1.684				
H	2.909	-2.281	0.469				
O	2.673	-6.962	1.896	W3_16	W14_2	W8_8	W3_3_4
H	2.508	-7.752	2.411				
H	2.700	-6.256	2.542				
O	1.400	-3.705	-3.691	W4_1	W4_1	W5_1	W4_1_1

H	0.786	-3.244	-3.120				
H	1.125	-4.621	-3.649				
O	-0.563	2.615	-5.084	W4_2	W6_3	W2_6	W4_4_3
H	-0.172	2.042	-5.743				
H	-1.351	2.152	-4.800				
O	2.730	3.782	-0.612	W4_3	W8_3	W6_8	W4_3_3
H	3.468	3.315	-1.003				
H	2.012	3.659	-1.232				
O	0.000	0.000	0.000	W4_4	W11_2	W5_2	W4_1_4
H	0.642	-0.179	0.687				
H	-0.705	-0.626	0.162				
O	-0.511	5.228	-2.373	W4_5	W1_4	W3_6	W4_2_2
H	-1.316	5.252	-2.891				
H	-0.326	4.296	-2.260				
O	3.401	1.372	-3.982	W4_6	W8_1	W6_7	W4_3_1
H	4.339	1.364	-4.169				
H	3.302	0.795	-3.224				
O	-0.030	0.356	-6.350	W4_7	W6_4	W2_1	W4_4_4
H	-0.733	-0.293	-6.328				
H	0.718	-0.114	-6.719				
O	-0.673	2.458	-0.951	W4_8	W11_4	W3_2	W4_2_3
H	-0.313	1.584	-0.795				
H	-0.379	2.974	-0.200				
O	5.584	2.372	-1.152	W4_9	W8_2	W6_1	W4_3_2
H	6.206	1.646	-1.107				
H	5.967	2.978	-1.787				
O	-2.859	0.856	-4.403	W4_10	W6_2	W2_8	W4_4_2
H	-3.688	1.135	-4.792				
H	-2.874	1.224	-3.519				
O	3.009	-2.142	-5.159	W4_11	W4_3	W5_5	W4_1_3
H	2.418	-2.701	-4.654				
H	3.511	-1.665	-4.498				
O	3.595	-0.268	-1.696	W4_12	W4_2	W5_4	W4_3_4
H	3.360	-0.757	-0.908				
H	4.268	0.347	-1.405				
O	-3.009	3.020	-2.402	W4_13	W1_2	W2_3	W4_2_4
H	-2.230	2.876	-1.864				
H	-3.209	3.949	-2.290				
O	1.034	-1.566	-2.036	W4_14	W4_4	W5_3	W4_1_2
H	0.903	-1.234	-1.147				
H	1.752	-1.039	-2.386				

O	1.397	2.991	-3.303	W4_15	W8_4	W6_3	W4_2_1
H	2.103	2.508	-3.733				
H	0.632	2.839	-3.857				
O	-2.361	-1.339	-6.316	W4_16	W6_1	W2_7	W4_4_1
H	-2.537	-0.700	-7.007				
H	-2.526	-0.863	-5.502				

Cluster 8

Element	X	Y	Z	hmbe_4_16	hmbe_16_4	hmbe_8_8	hmbe_4_4_4
O	3.016	4.017	0.056	W1_1	W16_3	W3_6	W1_1_1
H	2.977	4.832	-0.445				
H	3.310	3.360	-0.575				
O	-4.338	5.393	-0.875	W1_2	W8_3	W6_4	W1_4_4
H	-3.422	5.633	-1.011				
H	-4.431	4.545	-1.310				
O	1.799	0.405	-3.663	W1_3	W16_4	W8_6	W1_2_3
H	1.087	0.668	-3.080				
H	2.256	1.221	-3.867				
O	-0.525	1.101	-2.205	W1_4	W13_3	W8_3	W1_3_2
H	-0.314	0.741	-1.344				
H	-0.793	0.342	-2.724				
O	-2.139	3.306	-5.511	W1_5	W13_2	W2_7	W1_3_3
H	-1.680	4.146	-5.503				
H	-2.691	3.342	-6.292				
O	1.301	5.109	-3.669	W1_6	W4_1	W6_6	W1_2_1
H	2.078	5.421	-3.204				
H	0.727	4.776	-2.979				
O	-2.807	7.221	1.508	W1_7	W8_2	W6_8	W1_4_3
H	-2.192	6.979	0.816				
H	-2.775	6.489	2.124				
O	-0.335	7.305	0.083	W1_8	W4_2	W6_1	W1_4_1
H	0.481	6.898	0.374				
H	-0.115	8.230	-0.029				
O	2.908	4.240	2.689	W1_9	W10_1	W3_2	W1_1_3
H	3.257	4.329	1.801				

H	2.120	4.782	2.692				
O	4.580	2.488	-2.097	W1_10	W16_1	W8_5	W1_2_2
H	5.317	2.826	-2.606				
H	4.524	1.564	-2.341				
O	-1.740	5.704	-1.894	W1_11	W4_3	W6_7	W1_4_2
H	-0.982	5.160	-2.109				
H	-1.405	6.345	-1.267				
O	0.216	3.669	-1.575	W1_12	W4_4	W6_2	W1_3_4
H	-0.028	2.796	-1.882				
H	0.242	3.590	-0.621				
O	2.678	2.758	-4.692	W1_13	W16_2	W8_1	W1_2_4
H	1.958	3.333	-4.435				
H	3.469	3.256	-4.487				
O	-2.976	2.195	-3.058	W1_14	W13_4	W2_3	W1_3_1
H	-2.147	1.813	-2.771				
H	-2.807	2.492	-3.952				
O	0.000	0.000	0.000	W1_15	W2_3	W8_8	W1_1_2
H	-0.678	0.405	0.541				
H	0.496	-0.549	0.607				
O	0.319	3.861	1.673	W1_16	W10_4	W6_5	W1_1_4
H	0.568	2.964	1.898				
H	1.111	4.242	1.293				
O	4.837	-1.896	-0.424	W2_1	W12_4	W8_7	W2_1_4
H	4.223	-2.250	-1.067				
H	5.358	-1.260	-0.915				
O	3.008	-2.381	6.561	W2_2	W14_2	W1_8	W2_2_4
H	3.262	-1.937	7.370				
H	3.834	-2.528	6.099				
O	1.611	2.128	4.708	W2_3	W10_3	W3_7	W2_4_1
H	2.170	2.750	4.241				
H	2.146	1.339	4.789				
O	1.983	-1.258	1.248	W2_4	W9_2	W1_4	W2_2_2
H	1.743	-1.746	2.035				
H	2.810	-1.650	0.966				
O	7.418	-0.563	2.510	W2_5	W5_2	W3_1	W2_1_3
H	7.510	-1.367	1.999				
H	6.597	-0.676	2.990				
O	1.994	-5.436	4.898	W2_6	W14_3	W1_6	W2_3_1
H	2.112	-4.958	4.078				
H	2.731	-5.162	5.444				
O	3.078	-4.400	2.523	W2_7	W14_4	W1_2	W2_3_2

H	3.906	-4.090	2.155				
H	2.428	-4.209	1.847				
O	5.011	4.424	4.262	W2_8	W10_2	W3_8	W2_4_2
H	4.325	4.270	3.613				
H	4.554	4.409	5.103				
O	2.094	-1.525	4.203	W2_9	W9_1	W1_3	W2_2_1
H	2.331	-2.006	4.996				
H	2.738	-0.820	4.149				
O	4.388	0.125	1.615	W2_10	W5_4	W3_5	W2_1_2
H	5.280	0.044	1.278				
H	3.829	-0.024	0.853				
O	6.598	-0.251	-1.629	W2_11	W5_1	W8_4	W2_1_1
H	7.041	0.434	-1.128				
H	6.469	0.132	-2.496				
O	-0.569	-1.710	3.651	W2_12	W6_4	W7_3	W2_2_3
H	0.356	-1.933	3.555				
H	-0.566	-0.850	4.072				
O	3.946	0.735	4.109	W2_13	W9_3	W3_3	W2_4_4
H	4.050	0.646	3.161				
H	4.792	1.064	4.413				
O	1.710	1.249	2.067	W2_14	W9_4	W3_4	W2_4_3
H	2.646	1.440	2.117				
H	1.664	0.374	1.682				
O	5.287	-4.052	1.155	W2_15	W5_3	W1_7	W2_3_3
H	5.162	-3.309	0.564				
H	5.538	-4.775	0.580				
O	4.257	-6.785	2.788	W2_16	W14_1	W1_1	W2_3_4
H	4.513	-7.036	1.900				
H	3.747	-5.984	2.673				
O	-3.074	-1.712	4.688	W3_1	W6_1	W4_6	W3_3_3
H	-2.226	-1.608	4.257				
H	-3.431	-2.523	4.324				
O	-4.040	2.373	3.447	W3_2	W15_2	W7_7	W3_1_1
H	-3.373	3.060	3.458				
H	-3.841	1.858	2.664				
O	-7.369	0.297	1.207	W3_3	W11_1	W4_4	W3_2_1
H	-7.403	-0.550	0.764				
H	-6.483	0.620	1.040				
O	-2.275	2.253	6.556	W3_4	W15_3	W7_5	W3_4_2
H	-3.017	1.866	7.021				
H	-2.608	3.087	6.225				

O	-5.901	1.212	5.514	W3_5	W15_4	W7_8	W3_4_4
H	-6.442	0.646	4.965				
H	-5.598	1.901	4.923				
O	-5.147	1.213	-2.044	W3_6	W13_1	W2_5	W3_2_4
H	-4.291	1.530	-2.330				
H	-5.768	1.618	-2.649				
O	-0.990	0.946	4.130	W3_7	W6_3	W7_6	W3_1_3
H	-0.047	1.111	4.127				
H	-1.286	1.265	4.982				
O	-3.829	-4.182	3.415	W3_8	W3_1	W4_1	W3_3_1
H	-4.461	-4.570	2.811				
H	-3.321	-4.925	3.741				
O	-7.369	-2.224	-0.055	W3_9	W11_4	W4_2	W3_3_2
H	-7.971	-2.957	0.074				
H	-6.500	-2.624	-0.055				
O	-6.923	2.956	-0.356	W3_10	W8_1	W2_6	W3_2_3
H	-6.350	2.544	-1.003				
H	-7.773	2.534	-0.485				
O	-0.968	-0.021	7.545	W3_11	W6_2	W7_4	W3_4_3
H	-1.377	-0.719	7.034				
H	-1.456	0.768	7.307				
O	-4.125	2.211	0.366	W3_12	W2_2	W2_2	W3_2_2
H	-3.520	1.592	0.774				
H	-4.476	1.740	-0.390				
O	-4.184	-1.862	1.912	W3_13	W11_3	W4_3	W3_3_4
H	-3.905	-2.444	2.620				
H	-4.690	-2.424	1.326				
O	-2.350	4.652	2.654	W3_14	W8_4	W6_3	W3_1_4
H	-1.409	4.566	2.801				
H	-2.546	4.014	1.968				
O	-2.754	0.447	1.855	W3_15	W2_4	W7_2	W3_1_2
H	-2.217	0.523	2.644				
H	-3.260	-0.356	1.986				
O	-4.096	-0.011	6.864	W3_16	W15_1	W7_1	W3_4_1
H	-4.563	0.529	6.226				
H	-3.813	-0.778	6.366				
O	3.415	-3.248	-2.167	W4_1	W12_1	W8_2	W4_3_4
H	4.112	-3.537	-2.756				
H	2.643	-3.175	-2.728				
O	-1.693	-1.176	-3.105	W4_2	W1_3	W5_7	W4_4_3
H	-2.305	-1.266	-3.835				

H	-1.425	-2.073	-2.906				
O	0.951	-4.917	0.857	W4_3	W3_3	W1_5	W4_1_2
H	0.522	-4.126	0.531				
H	0.231	-5.481	1.141				
O	0.620	-4.049	-6.168	W4_4	W7_1	W5_1	W4_3_3
H	0.734	-4.940	-5.837				
H	0.759	-3.489	-5.404				
O	-1.431	-5.775	-3.520	W4_5	W7_4	W5_8	W4_2_2
H	-2.154	-5.410	-4.030				
H	-1.133	-6.528	-4.031				
O	-3.975	-4.872	-2.570	W4_6	W7_2	W5_5	W4_2_4
H	-3.262	-4.256	-2.737				
H	-4.092	-5.335	-3.400				
O	-5.478	-2.027	-4.659	W4_7	W1_4	W2_1	W4_4_4
H	-4.746	-1.676	-5.166				
H	-5.168	-2.022	-3.754				
O	-1.674	-3.063	0.828	W4_8	W2_1	W4_8	W4_1_3
H	-2.090	-3.411	1.616				
H	-2.158	-2.260	0.635				
O	-0.053	-7.184	-1.190	W4_9	W3_2	W5_2	W4_2_1
H	0.691	-6.815	-1.667				
H	-0.765	-6.561	-1.338				
O	-1.464	-6.150	2.140	W4_10	W3_4	W4_7	W4_1_4
H	-1.580	-6.177	3.090				
H	-2.337	-6.318	1.786				
O	2.103	-6.347	-2.967	W4_11	W12_2	W5_4	W4_3_1
H	2.146	-6.278	-3.921				
H	2.545	-5.560	-2.649				
O	-4.644	-1.440	-1.857	W4_12	W1_2	W2_4	W4_4_2
H	-5.210	-0.691	-1.673				
H	-3.779	-1.058	-2.005				
O	-0.868	-3.410	-2.006	W4_13	W7_3	W5_3	W4_2_3
H	-0.800	-4.305	-2.338				
H	-0.993	-3.513	-1.062				
O	1.161	-2.536	-3.673	W4_14	W12_3	W5_6	W4_3_2
H	1.349	-1.622	-3.888				
H	0.562	-2.488	-2.928				
O	-4.730	-3.536	-0.353	W4_15	W11_2	W4_5	W4_1_1
H	-4.723	-2.830	-0.999				
H	-4.471	-4.315	-0.846				
O	-3.167	-1.553	-5.386	W4_16	W1_1	W2_8	W4_4_1

H	-3.196	-0.845	-6.029
H	-2.628	-2.229	-5.798

Cluster 9

Element	X	Y	Z	hmbe_4_16	hmbe_16_4	hmbe_8_8	hmbe_4_4_4
O	-0.322	-0.029	-6.443	W1_1	W16_4	W4_8	W1_2_1
H	-0.179	-0.091	-5.498				
H	-1.222	0.283	-6.530				
O	3.716	-1.875	-3.058	W1_2	W9_3	W5_6	W1_4_3
H	3.496	-1.000	-2.739				
H	3.511	-2.456	-2.326				
O	3.748	2.277	-4.566	W1_3	W16_3	W4_6	W1_1_1
H	3.552	1.612	-5.226				
H	4.340	2.886	-5.008				
O	-0.373	-0.834	-3.745	W1_4	W3_3	W4_3	W1_2_4
H	-0.079	-1.733	-3.891				
H	-1.327	-0.880	-3.811				
O	-4.903	2.425	-5.819	W1_5	W10_2	W8_8	W1_3_2
H	-4.005	2.473	-6.147				
H	-5.178	1.527	-6.005				
O	2.382	0.885	-6.907	W1_6	W16_1	W4_1	W1_4_1
H	2.700	0.074	-6.511				
H	1.585	0.629	-7.371				
O	-2.283	4.319	-3.861	W1_7	W6_1	W8_3	W1_3_3
H	-1.365	4.558	-3.729				
H	-2.379	3.480	-3.410				
O	6.406	3.450	-2.047	W1_8	W12_3	W5_1	W1_1_2
H	6.549	2.928	-2.837				
H	5.607	3.945	-2.228				
O	0.711	-3.515	-5.243	W1_9	W3_4	W4_4	W1_2_2
H	0.926	-4.313	-4.760				
H	0.137	-3.810	-5.950				
O	0.208	3.203	-7.670	W1_10	W16_2	W4_5	W1_3_1
H	0.290	2.993	-8.600				
H	1.073	3.016	-7.305				

O	0.544	1.157	-2.421	W1_11	W6_2	W4_2	W1_2_3
H	1.430	0.970	-2.111				
H	0.325	0.412	-2.982				
O	6.334	-1.469	-4.658	W1_12	W9_2	W5_5	W1_4_2
H	5.865	-1.281	-5.471				
H	5.682	-1.891	-4.097				
O	3.182	3.954	-2.130	W1_13	W12_1	W1_2	W1_1_4
H	3.168	4.768	-2.634				
H	3.260	3.265	-2.790				
O	2.961	-1.570	-5.804	W1_14	W3_1	W4_7	W1_4_4
H	2.419	-2.356	-5.732				
H	3.234	-1.382	-4.906				
O	3.115	0.836	-2.091	W1_15	W12_2	W5_7	W1_1_3
H	3.723	0.554	-1.407				
H	3.676	1.214	-2.768				
O	-2.999	0.207	-6.199	W1_16	W10_4	W8_1	W1_3_4
H	-2.753	1.105	-6.421				
H	-3.480	-0.107	-6.965				
O	0.683	4.827	3.585	W2_1	W2_3	W7_4	W2_3_3
H	0.195	5.049	2.791				
H	0.765	3.874	3.557				
O	-2.599	2.196	-1.879	W2_2	W6_4	W8_7	W2_4_3
H	-2.724	1.249	-1.824				
H	-1.649	2.310	-1.926				
O	-4.048	2.450	3.698	W2_3	W14_3	W6_2	W2_1_3
H	-3.254	2.077	4.081				
H	-3.921	2.366	2.753				
O	-0.095	7.887	1.073	W2_4	W13_1	W1_1	W2_1_1
H	-0.815	8.216	0.534				
H	-0.061	6.950	0.880				
O	-3.783	6.197	3.160	W2_5	W7_2	W6_4	W2_1_2
H	-3.583	6.095	4.091				
H	-3.101	5.697	2.712				
O	3.273	5.465	1.108	W2_6	W13_2	W1_4	W2_2_1
H	3.519	4.543	1.023				
H	3.895	5.931	0.550				
O	-2.843	1.917	1.232	W2_7	W7_4	W6_5	W2_4_4
H	-2.594	2.830	1.092				
H	-3.494	1.738	0.553				
O	0.721	3.710	-1.409	W2_8	W4_1	W1_8	W2_2_2
H	0.525	2.975	-1.990				

H	1.602	3.986	-1.659				
O	0.411	2.532	6.544	W2_9	W2_1	W7_1	W2_3_1
H	0.043	3.186	7.138				
H	0.665	3.030	5.767				
O	-5.157	3.291	-2.714	W2_10	W6_3	W8_5	W2_4_1
H	-4.698	2.488	-2.960				
H	-5.024	3.364	-1.769				
O	-0.167	2.194	3.409	W2_11	W4_2	W7_3	W2_3_4
H	-0.777	1.485	3.614				
H	0.549	1.766	2.940				
O	-2.217	4.817	1.158	W2_12	W7_3	W1_5	W2_1_4
H	-1.336	4.555	0.888				
H	-2.558	5.313	0.415				
O	4.015	4.318	3.719	W2_13	W2_2	W7_5	W2_3_2
H	4.652	3.681	3.397				
H	3.650	4.714	2.927				
O	-5.159	3.362	0.032	W2_14	W7_1	W6_7	W2_4_2
H	-5.788	3.426	0.751				
H	-4.489	4.012	0.240				
O	0.607	5.183	0.925	W2_15	W13_3	W1_3	W2_2_4
H	1.519	5.446	1.044				
H	0.605	4.685	0.107				
O	1.687	7.389	-1.220	W2_16	W13_4	W1_7	W2_2_3
H	1.115	8.061	-1.589				
H	1.847	7.677	-0.321				
O	-5.078	-3.015	-1.984	W3_1	W1_4	W3_8	W3_1_1
H	-4.887	-3.953	-1.988				
H	-4.252	-2.601	-2.235				
O	-1.317	-7.243	1.339	W3_2	W11_3	W2_1	W3_3_3
H	-0.768	-6.477	1.171				
H	-2.213	-6.925	1.226				
O	-1.219	-0.794	2.254	W3_3	W14_2	W2_2	W3_2_2
H	-0.960	-0.837	1.334				
H	-2.007	-0.250	2.257				
O	-6.375	-0.022	-1.437	W3_4	W1_3	W6_8	W3_1_2
H	-6.605	-0.732	-0.838				
H	-7.208	0.410	-1.627				
O	-0.298	-2.417	-0.905	W3_5	W15_4	W3_4	W3_4_3
H	0.464	-2.717	-0.409				
H	-0.756	-3.221	-1.150				
O	-3.904	-5.664	1.855	W3_6	W11_1	W3_3	W3_3_1

H	-3.906	-5.646	0.898				
H	-4.818	-5.818	2.094				
O	-4.985	-1.056	4.939	W3_7	W14_4	W6_1	W3_2_3
H	-5.836	-1.241	4.541				
H	-4.854	-0.118	4.800				
O	-3.000	-2.071	-3.756	W3_8	W10_3	W8_6	W3_1_3
H	-3.168	-2.950	-4.096				
H	-3.131	-1.494	-4.508				
O	0.918	-5.369	-3.123	W3_9	W3_2	W3_6	W3_4_1
H	1.061	-6.276	-2.855				
H	0.051	-5.151	-2.783				
O	-3.402	-4.831	-4.093	W3_10	W10_1	W3_1	W3_4_2
H	-3.538	-5.762	-3.913				
H	-3.478	-4.757	-5.044				
O	-6.398	-2.171	0.500	W3_11	W1_1	W6_6	W3_2_1
H	-6.342	-2.692	-0.302				
H	-6.042	-2.741	1.181				
O	-3.106	-0.687	-1.132	W3_12	W1_2	W8_4	W3_1_4
H	-2.442	-1.318	-1.412				
H	-3.928	-1.037	-1.474				
O	-4.404	-5.705	-0.896	W3_13	W11_4	W3_7	W3_3_4
H	-5.345	-5.695	-1.072				
H	-4.120	-6.580	-1.160				
O	-0.618	-4.623	1.150	W3_14	W15_1	W2_8	W3_3_2
H	-1.373	-4.209	1.567				
H	0.111	-4.031	1.331				
O	-2.075	-4.281	-1.707	W3_15	W11_2	W3_5	W3_4_4
H	-2.495	-4.139	-2.555				
H	-2.669	-4.866	-1.239				
O	-6.081	0.335	1.489	W3_16	W14_1	W6_3	W3_2_4
H	-6.293	-0.588	1.349				
H	-5.490	0.556	0.769				
O	2.474	0.996	4.400	W4_1	W2_4	W7_8	W4_2_2
H	2.505	1.874	4.779				
H	2.241	1.139	3.482				
O	2.693	-5.961	0.573	W4_2	W15_2	W2_6	W4_3_1
H	2.247	-5.554	1.315				
H	2.227	-6.786	0.436				
O	4.769	-0.164	-0.319	W4_3	W5_4	W5_3	W4_1_3
H	5.701	0.036	-0.238				
H	4.674	-1.032	0.074				

O	1.729	1.387	1.784	W4_4	W4_4	W7_7	W4_1_4
H	1.109	1.103	1.112				
H	2.478	1.724	1.293				
O	-1.167	-2.930	6.158	W4_5	W8_4	W2_7	W4_4_2
H	-0.600	-3.514	6.662				
H	-2.008	-2.961	6.614				
O	1.107	-5.095	4.533	W4_6	W8_1	W2_5	W4_4_1
H	0.490	-4.509	4.095				
H	1.837	-4.529	4.785				
O	6.442	-1.313	2.613	W4_7	W5_3	W5_4	W4_2_3
H	7.284	-0.944	2.346				
H	5.895	-0.551	2.801				
O	6.050	-3.741	-2.129	W4_8	W9_1	W5_2	W4_3_2
H	6.060	-4.147	-1.262				
H	5.590	-2.911	-2.000				
O	4.402	-2.467	1.133	W4_9	W5_2	W5_8	W4_3_3
H	4.894	-1.937	1.760				
H	4.780	-3.343	1.210				
O	0.000	0.000	0.000	W4_10	W4_3	W8_2	W4_1_2
H	0.163	0.562	-0.758				
H	-0.077	-0.881	-0.366				
O	1.708	-3.114	1.707	W4_11	W15_3	W2_3	W4_4_3
H	2.660	-3.051	1.778				
H	1.380	-2.718	2.515				
O	0.155	-2.355	3.971	W4_12	W8_2	W2_4	W4_4_4
H	-0.375	-2.394	4.767				
H	-0.354	-1.811	3.370				
O	2.788	-1.560	5.097	W4_13	W8_3	W7_6	W4_2_4
H	2.900	-0.636	4.876				
H	2.100	-1.866	4.505				
O	2.540	-3.865	-1.117	W4_14	W9_4	W3_2	W4_3_4
H	2.175	-4.203	-1.935				
H	2.664	-4.641	-0.571				
O	5.242	0.905	3.894	W4_15	W5_1	W7_2	W4_2_1
H	4.365	1.275	3.999				
H	5.485	0.610	4.772				
O	3.508	2.633	0.288	W4_16	W12_4	W1_6	W4_1_1
H	4.223	2.037	0.063				
H	3.213	2.982	-0.553				
